# Connexin 32-mediated cell-cell communication is essential for hepatic differentiation from human embryonic stem cells

**DOI:** 10.1038/srep37388

**Published:** 2016-11-22

**Authors:** Jinhua Qin, Mingyang Chang, Shuyong Wang, Zhenbo Liu, Wei Zhu, Yi Wang, Fang Yan, Jian Li, Bowen Zhang, Guifang Dou, Jiang Liu, Xuetao Pei, Yunfang Wang

**Affiliations:** 1 Stem Cell and Regenerative Medicine Lab, Beijing Institute of Transfusion Medicine, Beijing 100850, China; 2 Tissue Engineering Lab, Beijing Institute of Transfusion Medicine, Beijing 100850, China; 3 South China Research Center for Stem Cell and Regenerative Medicine, South China Institute of Biomedicine, Guangzhou 510005, China; 4 CAS Key Laboratory of Genome Sciences and Information, Beijing Institute of Genomics, Chinese Academy of Sciences, Beijing 100101, China; 5 Laboratory of Hematological Pharmacology, Beijing Institute of Transfusion Medicine, Beijing 100850, China

## Abstract

Gap junction-mediated cell-cell interactions are highly conserved and play essential roles in cell survival, proliferation, differentiation and patterning. We report that Connexin 32 (Cx32)-mediated gap junctional intercellular communication (GJIC) is necessary for human embryonic stem cell-derived hepatocytes (hESC-Heps) during step-wise hepatic lineage restriction and maturation. Vitamin K2, previously shown to promote Cx32 expression in mature hepatocytes, up-regulated Cx32 expression and GJIC activation during hepatic differentiation and maturation, resulting in significant increases of hepatic markers expression and hepatocyte functions. In contrast, negative Cx32 regulator 2-aminoethoxydiphenyl borate blocked hESC-to-hepatocyte maturation and muted hepatocyte functions through disruption of GJIC activities. Dynamic gap junction organization and internalization are phosphorylation-dependent and the p38 mitogen-activated protein kinases pathway (MAPK) can negatively regulate Cxs through phosphorylation-dependent degradation of Cxs. We found that p38 MAPK inhibitor SB203580 improved maturation of hESC-Heps correlating with up-regulation of Cx32; by contrast, the p38 MAPK activator, anisomycin, blocked hESC-Heps maturation correlating with down-regulation of Cx32. These results suggested that Cx32 is essential for cell-cell interactions that facilitate driving hESCs through hepatic-lineage maturation. Regulators of both Cx32 and other members of its pathways maybe used as a promising approach on regulating hepatic lineage restriction of pluripotent stem cells and optimizing their functional maturation.

The liver is the major organ responsible for protein synthesis, metabolic transformation, and detoxification of xenobiotics as well as for metabolically handling endogenous substrates. The hepatocyte is the most important cell type for both cell therapy and liver regeneration for end-stage liver diseases and for toxicity evaluation during drug development in pharmaceutical industries^1^,^2^. However, primary human hepatocytes (PHH) are a severely limited resource given the shortage of donor livers. They cannot easily be expanded, and they lose their metabolic functions rapidly *in vitro*^3^,^4^. With pluripotency and unlimited self-renewal capacity, human embryonic stem cells (hESCs) and induced pluripotent stem cells (hiPSCs) could become an effective source of human hepatocytes for clinical and industrial applications^5^,^6^,^7^,^8^. Deriving of hepatocytes from either hESCs or hiPSCs have been attempted numerously until now^6^,^9^,^10^,^11^. However, hESC/iPSC-derived hepatocytes usually carried fetal hepatocyte-like phenotype and functions^12^, which indicated that maturation of these derived hepatic cells *in vitro* was a popular problem and one of the major challenges in research. Therefore, new experimental strategies are expected to achieve a successful differentiation of fully mature hepatocytes from pluripotent stem cells.

Gap junctions are the pores coupling adjacent cells to mediate intercellular activities of gap junctional intercellular communication (GJIC), by which there is exchange of metabolites and electrical activity^13^. They are formed by connexons, “iris-diaphragm-like” structures composed of 6 connexins (Cxs) that can assume a closed position forming a small channel, or swivel open to form a larger channel. The Cxs comprise a large family of proteins and most cell types express more than one type of Cx. Both Cx expression and GJIC activity may vary with physiological and pathological states of the cell and tissue. The gap junctional exchange of small molecules between adjacent cells is crucial for maintaining tissue homeostasis^14^. Importantly, genetic mutations in Cx interfered with GJ function resulting in several diseases^15^,^16^,^17^. It was also suggested that GJIC and Cxs played critical roles in stem cell proliferation and differentiation. Schiller *et al.* showed that inhibition of GJIC blocked the progression of pre-osteoblastic cells towards a mature, osteoblastic phenotype *in vitro*, but favored an adipocytic phenotype^18^. Li *et al.* deduced that modulation of Cx43 altered expression of osteoblastic differentiation markers^19^. On the other hand, increasing Cx43 expression by the treatment of all-trans retinoic acid resulted in more differentiation and maturation of lens epithelial cells^20^. Furthermore, Cx43 overexpression potentiated and induced dentin sialophosphoprotein expression and enhanced odontoblastic differentiation of dental pulp stem cells^21^.

Multiple forms of Cxs, including Cx26 and Cx32, were found in hepatic parenchymal cells in adult livers. There are ~90% Cx32 and ~5% Cx26 in well-organized tissue of adult liver, which establish an elaborate GJIC network between hepatocytes and become indispensable for functional differentiation^22^. In adult liver, Cx32 expression and GJIC activities positively correlate with CYP-mediated xenobiotic biotransformation^23^,^24^,^25^, glycogenolysis^26^,^27^, albumin secretion^28^, ammonia detoxification^28^ and bile secretion^29^.

More importantly, Cx expression patterns in embryonic liver undergo lineage stage-dependent changes during hepatic differentiation and maturation process. Hepatic progenitor cells were indeed repeatedly found to switch from Cx43 to Cx26 expression and, in particular, to Cx32 expression upon differentiation into hepatocytes, both *in vivo*^22^,^30^,^31^ and *in vitro*^31^,^32^. The natural compound Vitamin K2 (VK2) from the gut microbiome has been reported to contribute to the maturation of pluripotent stem cells to hepatocytes^10^. Previous data suggested that VK2 could up-regulate Cx32 at the transcriptional level^33^. Taken together, those findings support a hypothesis that Cx32 and its regulation might be critical for maturation of hESC-Heps.

The p38 MAPK pathway has been shown to negatively regulate the expression, formation and function of Cxs-mediated gap junctions through phosphorylation-dependent degradation of Cxs^34^. p38 inhibitor, SB203580 (SB) can protect the mouse from partial hepatectomy, the mechanism is likely to be that SB inhibited the down-regulation of Cx32 resulting in the maintenance of the structure of gap junctions^35^. Given the correlation between Cx32 expression and hepatocyte functional maintenance, we hypothesized that enhanced hepatocyte maturity might be achieved by modulating Cx32 directly or indirectly in the process of hESCs’ hepatic differentiation by those regulators we mentioned above.

In this study, we show that during the three-stage differentiation protocol to produce hESC-Heps, both Cx32’s activator VK2 and p38-MAPK inhibitor SB can enhance the expression of various hepatic markers and improve hepatic functions of hESC-Heps through improvements of Cx32 expression and GJIC activities. On the other hand, inhibition of Cx32 or activation of p38 results in poor hepatic differentiation. Our work suggests that Cx32-mediated cell-cell communications play a critical role in hepatic lineage restriction and maturation of hESCs to hepatocytes, and improved Cx32 expression and GJIC activity can enhance hepatocyte functional maturity.

## Results

### Lineage restriction and maturation of hESCs to hepatocytes

**Figure 1:**
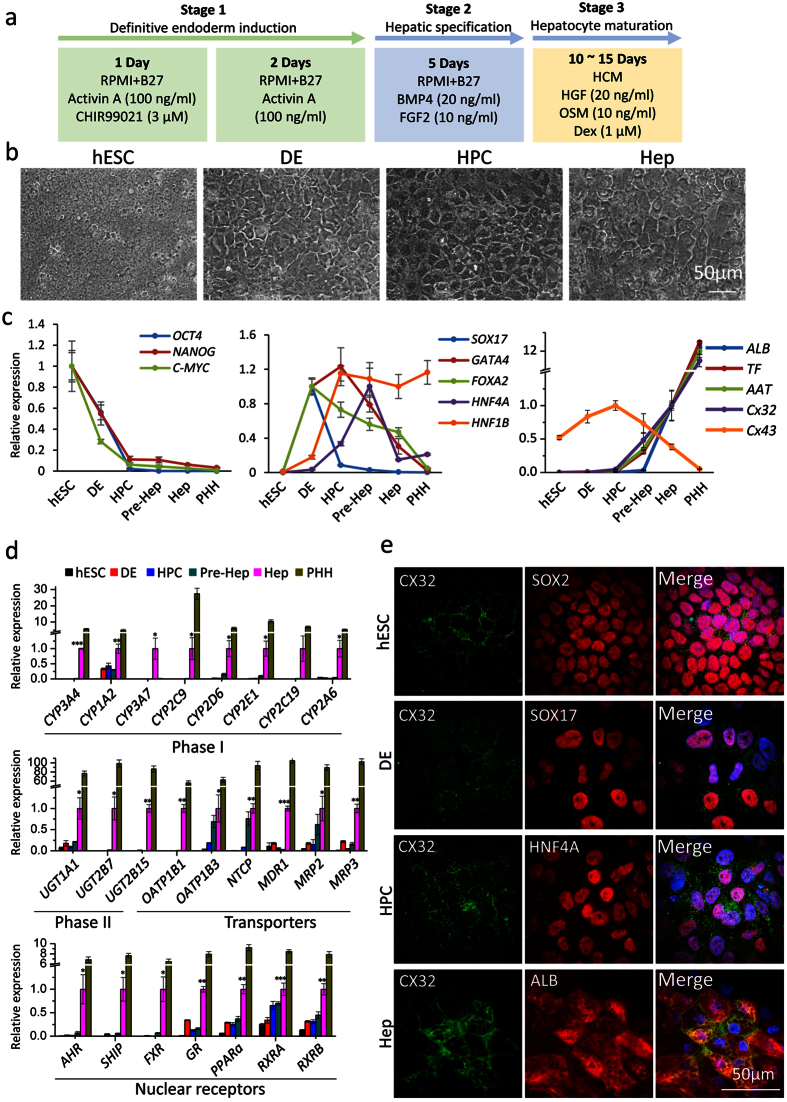
Lineage restriction of human ESCs to hepatocytes. (**a**) Schematic description of the three-stage protocol used to achieve differentiation of hESCs to hepatocytes. (**b**) Representative morphology of cells observed at key stages of differentiation using phase contrast microscopy. (**c**) Quantitative reverse-transcriptase polymerase chain reaction (qRT-PCR) analysis of transcription factors maintaining pluripotency (*OCT4*, *NANOG* and *C-MYC*), transcription factors regulating hepatic differentiation (*SOX17*, *GATA4*, *FOXA2*, *HNF4A*, and *HNF1B*), key liver proteins (*ALB*, *TF*, *AAT*), and gap junction proteins (*Cx32* and *Cx43*). Pluripotency factors disappear by the DE stage, followed by a transient expression of early endodermal genes and the gradual appearance of hepatocyte-specific proteins. hESC, human embryonic stem cell; DE, definitive endoderm; HPC, hepatic progenitor cell; Pre-Hep, the early stage of hepatocyte-like cells at about day 15; Hep, hepatocytes derived from hESCs at about day 25. PHH, primary human hepatocytes. (**d**) mRNA expression of drug metabolism phase I and phase II enzymes, phase III transporters, and nuclear receptors. (**e**) Immunostaining of Cx32 and stage-specific makers of cells at different stages of differentiation. Scale bars, 50 μm. Data represent mean ± SEM. *P < 0.05, **P < 0.01, ***P < 0.001.

Based on previous studies, a stepwise 3-stage protocol was used to induce hESCs differentiation to hepatocytes (hESC-Heps) (Fig. 1a). More than 95% of hESCs expressed pluripotency markers, including SRY (sex determining region Y)-box 2 (SOX2) and octamer-binding transcription factor 4 (OCT4) (Fig. 1b,c and Supplementary Fig. S1). SOX17-positive definitive endoderm (DE) was efficiently induced in the first three days during stage 1, in which forkhead box protein A2 (FOXA2) and GATA-binding protein 4 (GATA4) were dramatically increased. In the next hepatic specification stage, expression of liver transcriptional factors, HNF4A and HNF1B, were initiated, while DE marker SOX17 was reduced and pluripotent markers SOX2, NANOG and OCT4 disappeared by day 8. In the final stage of differentiation (hepatocyte maturation), high levels of alpha-fetoprotein (AFP) were expressed in the early stage of formation of hepatocyte-like cells (Pre-Hep) at about day 15; cells were found to express high levels of mature hepatocyte markers, such as albumin (ALB) and cytokeratin 18 (CK18) at about day 25 (Fig. 1b,c and Supplementary Fig. S1). Meanwhile, many functional genes of hepatocytes, including transferrin (*TF*), alpha-1-anti-trypsin (*AAT*), drug metabolism phase I and phase II enzymes, phase III transporters, and detoxification-related nuclear receptors, were significantly increased in hepatocytes derived from hESCs (Fig. 1c,d). Importantly, expression of Cx32 correlated with acquisition of mature functions, and the localization of Cx32 changed from cytoplasm to membrane during the differentiation process (Fig. 1d,e). This protocol produced similar results in the H1 hESC cell line (Supplementary Fig. S3a).

### Cx32 regulators affected the expression of hepatic markers in hESC-Heps

To explore the effects of Cx32 on hepatocyte differentiation, VK2 and 2-aminoethoxydiphenyl borate (2-APB), which have been shown to directly increase^33^ or inhibit^36^ Cx32 gap junctions *in vitro,* respectively and effectively improve^33^ or block^37^ hepatic gap junction communication *in vivo*, were used to test Cx32 relevance. When added to the last stage of differentiation, VK2 caused a dose-dependent induction of *Cx32*, *ALB* and *AAT* expression. *Cx32* was induced about 3-fold by VK2 at 50 μM (Supplementary Fig. S2a). In contrast, addition of 2-APB to the last stage of differentiation caused reduction of these genes, and down-regulated *Cx32* by 3-fold at 50 μM (Supplementary Fig. S2b). Therefore, subsequent differentiation was carried out at 50 μM of VK2 and 2-APB.

**Figure 2:**
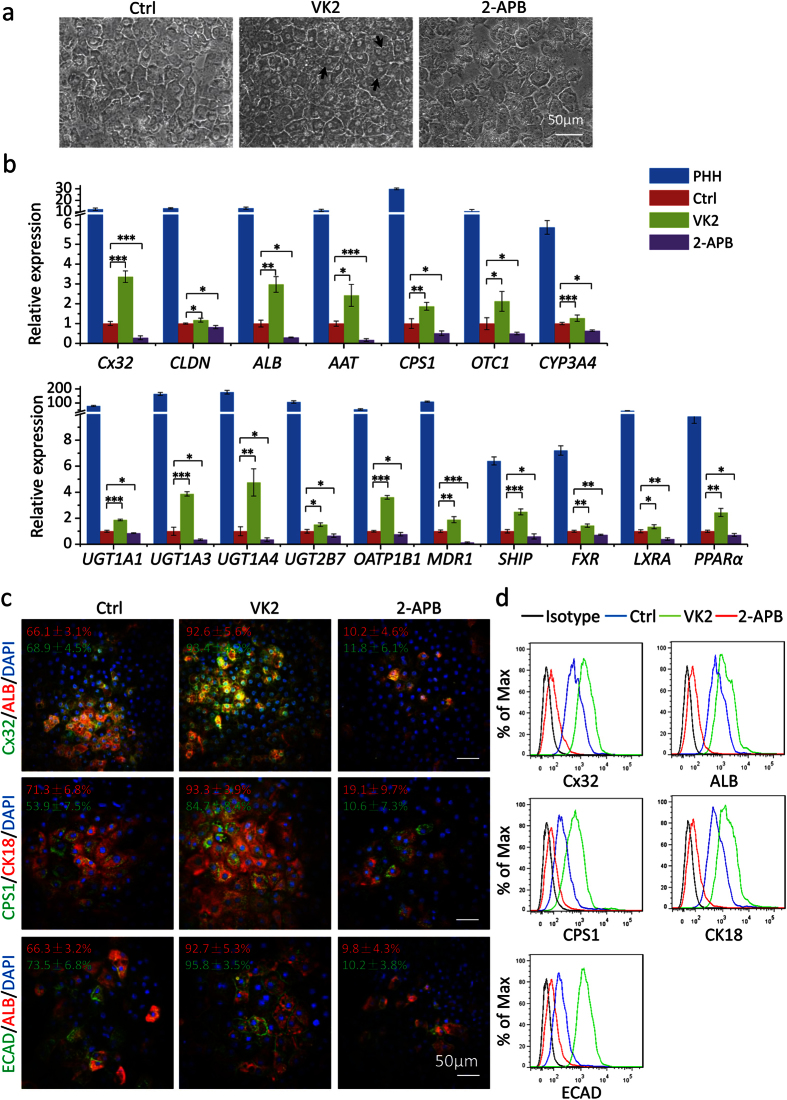
Expression of hepatic markers in hESC-Heps induced with VK2 and 2-APB. (**a**) Representative morphology of hESC-Heps cultured with VK2 or 2-APB during the final stage of differentiation. (**b**) qRT-PCR analysis of hESC-Heps induced with VK2 or 2-APB. (**c**) Immunostaining of Cx32, ALB, CPS1, CK18 and ECAD in hESC-Heps induced with VK2 or 2-APB. (**d**) Flow cytometry analysis of Cx32, ALB, CPS1, CK18 and ECAD in hESC-Heps induced with VK2 or 2-APB. Scale bars, 50 μm. Data represent mean ± SEM. *P < 0.05, **P < 0.01, ***P < 0.001.

By day 20 of differentiation, cells induced with the treatment of VK2 were large and homogeneously polygonal shaped with bright junctions. A small fraction became binucleated (arrows), and these displayed more typical hepatocyte morphology than cells in DMSO-treated control group (Fig. 2a). To compare the gene expression of the hepatocytes induced under these conditions, a repertoire of hepatic markers were analyzed by qRT-PCR. These included plasma proteins (*ALB* and *AAT*) and genes related to cell-cell communication (*Cx32* and *CLDN1*), the urea cycle (*CPS1* and *OTC1*), drug metabolism phase I enzymes (*CYP3A4*), phase II enzymes (*UGT1A1*, *UGT1A3*, *UGT1A4* and *UGT2B7*), phase III transporters (*OATP1B1* and *MDR1*), and hepatic nuclear receptors (*SHIP*, *FXR*, *LXRA* and *PPARα*). The observed relative levels of expression were similarly higher in hESC-Heps treated with VK2 than untreated cells (Fig. 2b). In addition, immunostaining and flow cytometry data showed that cells treated with VK2 demonstrated more homogeneous and enhanced expression of ALB, Cx32, CK18, CPS1 and E-cadherin (ECAD) than untreated cells (Fig. 2c,d and Supplementary Fig. S4). In our previous study, we found that overexpression of Cx32 in hepatic progenitor-like cells WB-F344 resulted in improved expression of hepatocyte markers when induced to hepatic differentiation (data not shown). These results indicated that enhanced expression of Cx32 by genomic or chemical induction could promote hepatocyte differentiation.

On the contrary, cells differentiated in the presence of 2-APB showed poorer morphology and looser contacts between cells with a higher nuclei/plasma ratio (Fig. 2a). Moreover, the gene expression of hepatic markers were lower in cells treated with 2-APB, and only several scattered cells were positively stained with these mature hepatic markers when induced with 2-APB (Fig. 2b–d and Supplementary Fig. S4). In addition, Cx32 was knocked-down in hESCs-derived hepatic progenitor cells using siRNA, and the expression levels of hepatocyte markers were dramatically decreased when the cells were further induced to differentiation (Supplementary Fig. S5). These results suggested that inhibition of Cx32 resulted in impaired hepatic differentiation, which further proved the vital role of Cx32 in hepatocyte differentiation.

### Inhibition of p38 MAPK pathway promoted hepatic differentiation of hESCs

**Figure 3:**
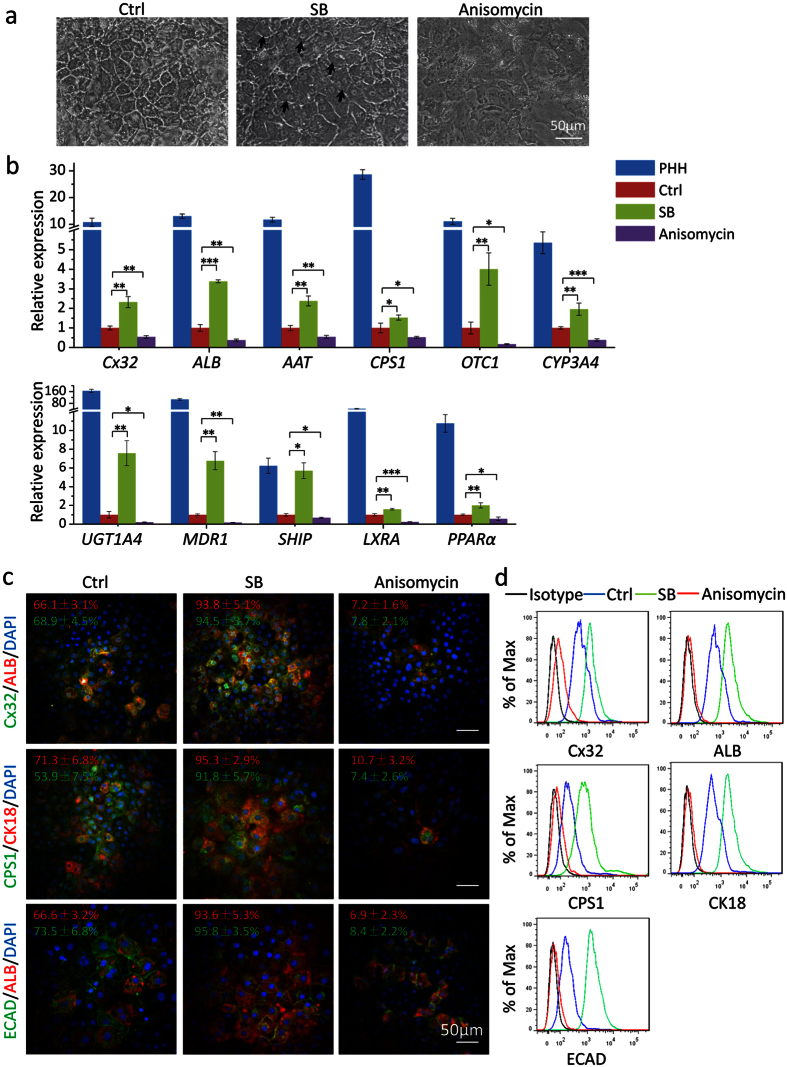
Expression of hepatic markers in hESC-Heps induced with SB and anisomycin. (**a**) Representative morphology of hESC-Heps cultured with SB or anisomycin during the final stage of differentiation. (**b**) qRT-PCR analysis of hESC-Heps induced with SB or anisomycin. (**c**) Immunostaining of Cx32, ALB, CPS1, CK18 and ECAD in hESC-Heps induced with SB or anisomycin. (**d**) Flow cytometry analysis of Cx32, ALB, CPS1, CK18 and ECAD in hESC-Heps induced with SB or anisomycin. Scale bars, 50 μm. Data represent mean ± SEM. *P < 0.05, **P < 0.01, ***P < 0.001.

p38 MAPK pathway was shown to regulate negatively the function of Cx32-mediated gap junctions^35^. We used a p38 MAPK inhibitor, SB, in the last step of hepatocyte differentiation, and observed more binucleated polygonal hepatocytes and an increased expression of hepatic markers than untreated controls (Fig. 3a). *Cx32*, *ALB* and *AAT* were induced about 3-fold by SB at 10 μM (Supplementary Fig. S2c). The expression of hepatic markers, including Cx32, ALB, AAT, OTC1, UGT1A4, MDR1, etc. were increased in hESC-Heps treated with SB than untreated cells (Fig. 3b). Additionally, immunostaining and flow cytometry data showed that cells treated with SB demonstrated more homogeneous and enhanced expression of ALB, Cx32, CK18, CPS1 and ECAD than untreated cells (Fig. 3c,d and Supplementary Fig. S4). On the contrary, anisomycin, an activator of p38 MAPK, disrupted hepatocyte differentiation and decreased expression of hepatic markers dramatically (Fig. 3b–d and Supplementary Fig. S4). This consistent outcome of treatment by VK2 which up-regulated Cx32 and SB which inhibited p38 MAPK were also observed in the hepatic differentiation of the H1 hESC cell line (Supplementary Fig. S3b).

To confirm these findings, fetal human hepatocytes (FHHs) isolated from fetuses at 16–20 weeks of gestation were differentiated with SB for 4 days and compared to DMSO-treated controls. Surprisingly, SB treatment drove expression of many mature hepatocyte-specific markers, including *Cx32*, *ALB*, *ASGR1*, *TAT* and *TF*, and suppressed expression of fetal hepatocyte genes, *AFP* and *Cx43*. In the meanwhile, functional genes involved in drug metabolism were improved by SB treatment, such as phase I and phase II enzymes, phase III transporters and detoxification-related nuclear receptors (Supplementary Fig. S6). These results confirmed that SB influences hepatic maturation.

### Effect of SB, VK2 and 2-APB on GJIC activity

**Figure 4:**
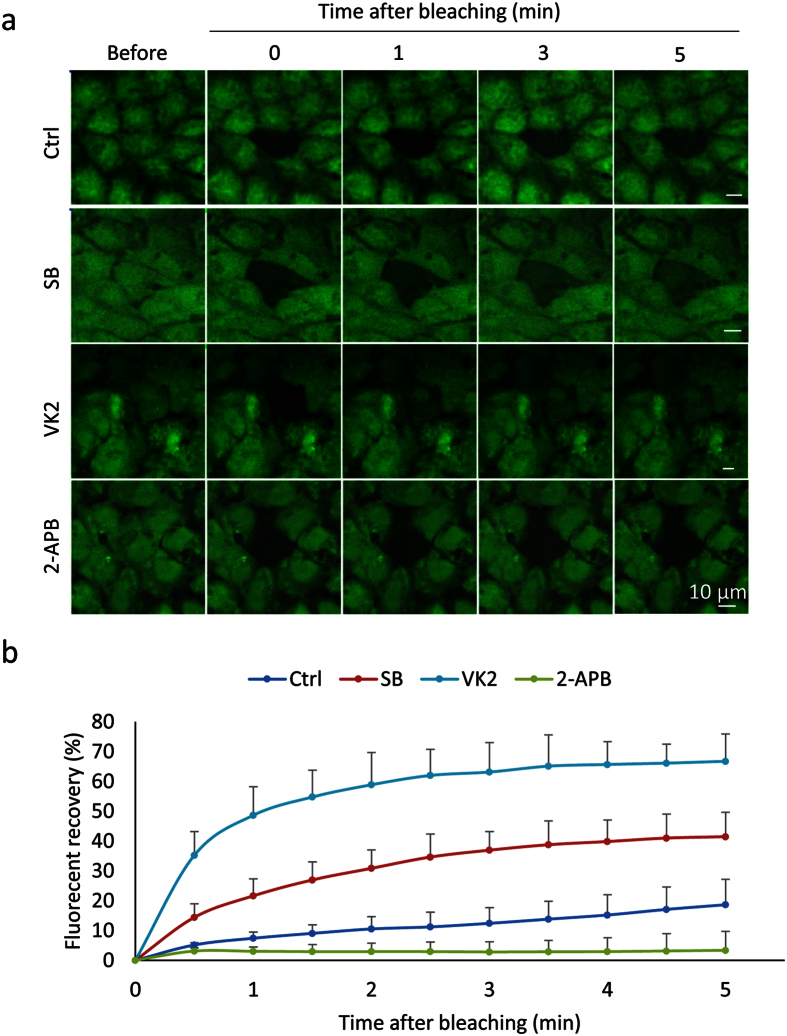
Effects of SB, VK2 and 2-APB on gap junction intercellular communication (GJIC). (**a**) Representative images of fluorescence recovery in bleached cells before bleaching, just after bleaching and 1, 3 and 5 min after bleaching. Scale bars, 10 μm. (**b**) Fluorescence recovery curves. The fluorescence intensity of an unbleached cell was considered as 100%. The intensity in the bleached cell in different groups were normalized. Each plot represents the averaged results from ten different cells. Differences between fluorescence recovery curves were estimated using two-way ANOVA (*P* < 0.001).

The establishment of an elaborate GJIC network between cells is important for hepatocyte differentiation. As we observed up-regulated expression of Cx32 which represented the major gap junction protein in hepatocytes, FRAP was used to evaluate the effect of SB, VK2 and 2-APB on GJIC activity of the hESC-Heps. The fluorescence recovery of representative cells before, just after and 1, 3, or 5 min after bleaching were recorded. Fluorescence recovery was significantly enhanced by both SB and VK2 treatment, while 2-APB blocked the recovery of fluorescence, as expected (Fig. 4a). Quantitative data demonstrated that hESC-Heps in the control group exhibited approximately 18% recovery at 5 min after bleaching. In contrast, the recovery rate was almost 2- and 3-fold faster with SB or VK2 treatment, respectively (Fig. 4b). Thus, SB and VK2 were able to improve the GJIC activity, and this was mediated primarily by Cx32 in hepatocytes differentiated from hESCs.

### Hepatocyte functions were susceptible to Cx32 regulators

**Figure 5:**
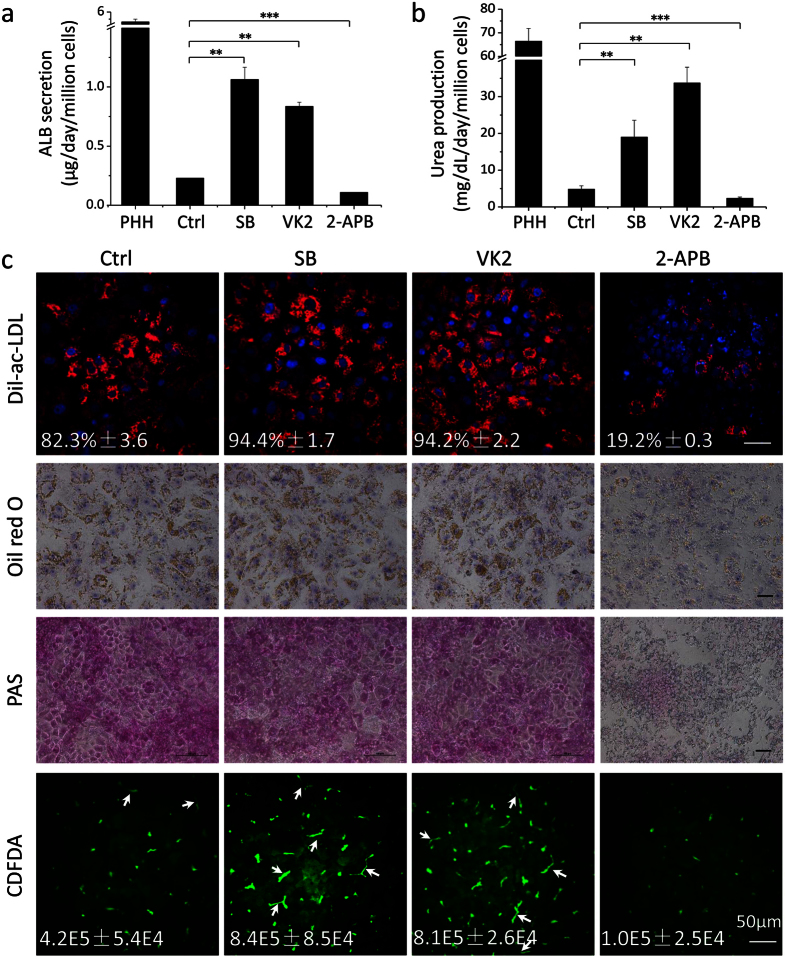
Effects of SB, VK2 and 2-APB on hepatocyte functions. (**a**) Albumin secretion of hESC-Heps induced with SB, VK2 or 2-APB. (**b**) Urea production of hESC-Heps induced with SB, VK2 or 2-APB. (**c**) Dil-ac-LDL uptake, oil red O staining, PAS staining, and CDFDA staining of hESC-Heps induced with SB, VK2 or 2-APB. Staining intensity was normalized to cell number. Scale bars, 50 μm. Data represent mean ± SEM. **P < 0.01, ***P < 0.001.

The secreted levels of ALB detected in the hESC-Heps culture supernatant were much higher in SB or VK2 groups than that in the control group, and were lower, as expected, in the 2-APB group (Fig. 5a). By day 25, the hESC-Heps induced with SB or VK2 produced urea at about 3 to 6 times higher than those in the control group, whereas 2-APB reduced urea production by one fold (Fig. 5b). PHHs were used as a positive control in the above quantitative assays. Additional functions included low density lipoprotein (LDL) uptake, cytoplasmic accumulation of neutral triglycerides and lipids, and glycogen storage (Fig. 5c). We observed consistently better results in SB and VK2 groups than those in the control group or 2-APB group.

Furthermore, bile canaliculi functions were evaluated by carboxydichlorofluroscein diacetate assays. The 5 (and 6)-Carboxy-2′,7′-dichlorofluorescein diacetate (CDFDA) readily diffused into cells, where it was metabolized to fluorescent CDF and excreted into the bile canaliculi by the multidrug resistance-associated protein 2 (MRP2). Of note, hESC-Heps supplied with SB or VK2 demonstrated denser CDF accumulation and more functional bile canaliculi (arrows) formation in comparison with the controls (no treatment group). However, 2-APB treatment resulted in even lower CDF accumulation and much less bile canaliculi formation (Fig. 5c).

**Figure 6:**
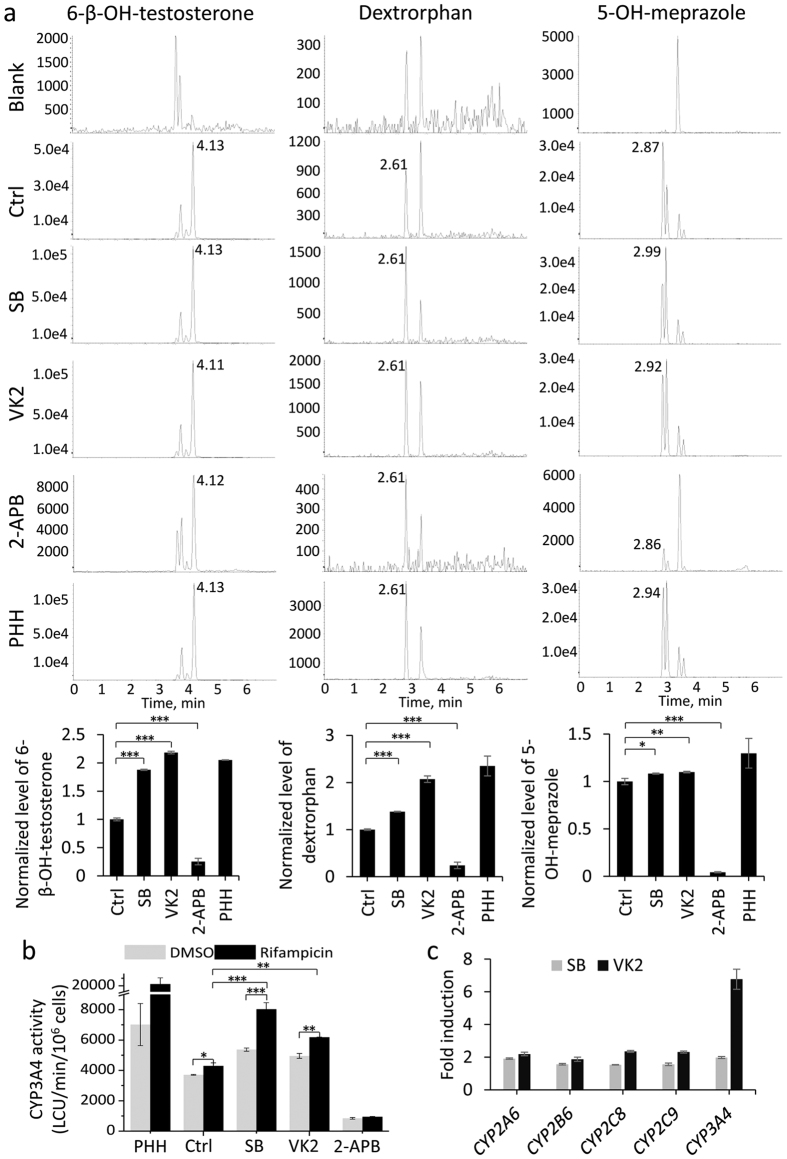
Effects of SB, VK2 and 2-APB on CYP450 activity. (**a**) CYP metabolic activity in hESC-Heps induced with SB, VK2 or 2-APB. The metabolic products of testosterone (6-β-OH-testosterone), dextromethorphan (dextrorphan), and omeprazole (5-OH-meprazole), assays for CYP3A4, 2D6 and 2C19 activities, respectively, were determined by liquid chromatography-tandem mass spectrometry. Data were calculated using area under the curve and normalized to hESC-Heps without treatment (controls). (**b**) Induction of CYP3A4 activity in response to 72 hours of stimulation with PXR agonist rifampicin. (**c**) The mRNA levels of the induced CYP enzymes in ESC-Heps treated with SB or VK2 were measured by qRT-PCR. Fold inductions were normalized to the levels in cells without inducer treatment, respectively. Data represent mean ± SEM. *P < 0.05, **P < 0.01, ***P < 0.001.

To evaluate CYP450 activity in hESC-Heps, we monitored the metabolism of testosterone, dextromethorhan and omeprazole, the substrates metabolized by CYP3A4, CYP2D6 and CYP2C19, respectively. Their respective major metabolites, 6-β-OH-testosterone, dextrorphan and 5-OH-meprazole were determined using high pressure liquid chromatography (HPLC). Normalized data showed that treatment with SB caused a 2- and 1.5-fold increase in testosterone and dextromethorphan metabolism respectively. VK2 induced about 2-fold increase in metabolism of both the two substrates, compared to untreated cells. In contrast, 2-APB severely reduced the activity to metabolize all three substrates (Fig. 6a). Furthermore, a sensitive and selective bioluminescent assay also confirmed CYP3A4 activity was improved by SB and VK2, and was inhibited by 2-APB (Fig. 6b). Importantly, the CYP3A4 activity in SB- or VK2- treated hESC-Heps were inducible by rifampicin (Fig. 6b). Furthermore, rifampicin treatment induced mRNA expression levels of *CYP3A4*, *CYP2A6*, *CYP2B6*, *CYP2C8* and *CYP2C9* in both SB and VK2 induced hepatocytes (Fig. 6c).

### SB and VK2 drive hepatic maturation

**Figure 7:**
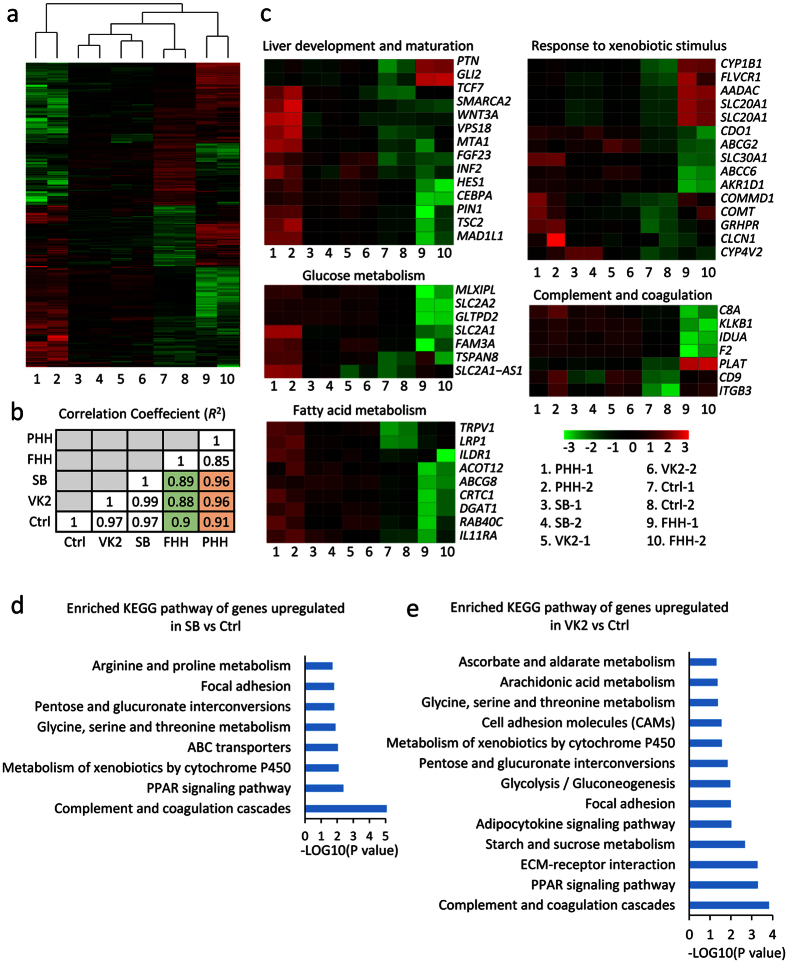
Global gene analysis of hESC-Heps induced with SB and VK2 by RNA-sequencing. (**a**) Heatmap of 707 genes differentially expressed between hESC-Heps induced with or without SB and VK2, with PHHs and FHHs as controls. (**b**) Correlation coefficients (R^2^) of the transcriptomes examined between different cell types. (**c**) Heatmaps of genes that exhibited significantly different expression levels among genes involved in liver development and maturation, glucose metabolism, fatty acid metabolism, drug metabolism, and complement and coagulation. (**d**) Selected enriched KEGG pathways of genes up-regulated in SB-induced hESC-Heps, compared to untreated controls. (**e**) Selected enriched KEGG pathways of genes up-regulated in VK2-induced hESC-Heps, compared to untreated controls.

To explore the extent of expression of characteristic fetal and mature hepatic markers, we carried out RNA sequencing (RNA-Seq) analysis on SB- and VK2-treated hESC-Heps, comparing them to untreated controls, PHHs and FHHs. The gene expression profile of SB- and VK2-induced hESC-Heps clustered closer to adult than that to fetal hepatocytes (Fig. 7a,b). Genes involved in regulation of liver development and maturation and in typical liver functions, including glucose metabolism, fatty acid metabolism, drug metabolism, and complement and coagulation, were up-regulated in SB- and VK2-treated hESC-Heps, compared to untreated controls as well as to FHHs (Fig. 7c). Furthermore, we analyzed 452 genes up-regulated in SB-treated hESC-Heps and 1427 genes up-regulated in VK2-treated hESC-Heps compared to controls by KEGG. We found that the enriched pathways included PPAR signaling pathway, metabolism of xenobiotics by CYP450, pentose and glucuronate inter-conversions, glycine, serine and threonine metabolism, complement and coagulation cascades and others (Fig. 7d,e). Taken together, these data suggested a global effect of SB and VK2 stimulation.

### Stabilization of Cx32-mediated GJ by inhibition of Cx32 phosphorylation

Pathway analysis of RNA-seq data showed that with SB-treatment, MAP kinase phosphatases (*DUSP4* and *DUSP9*) which inhibit the p38 pathway were up-regulated (Supplementary Fig. S7a). Similarly, under the treatment of VK2, MAP kinase phosphatases (*DUSP8*, *DUSP9* and *DUSP14*), as well as a protein tyrosine phosphatase (*PTPN7*), which is also an inhibitor for the p38 pathway, were up-regulated (Supplementary Fig. S7b). The bioinformatics data suggested that both SB and VK2 might affect the hepatic differentiation process in similar ways through negative regulation of the p38 signaling pathway.

**Figure 8:**
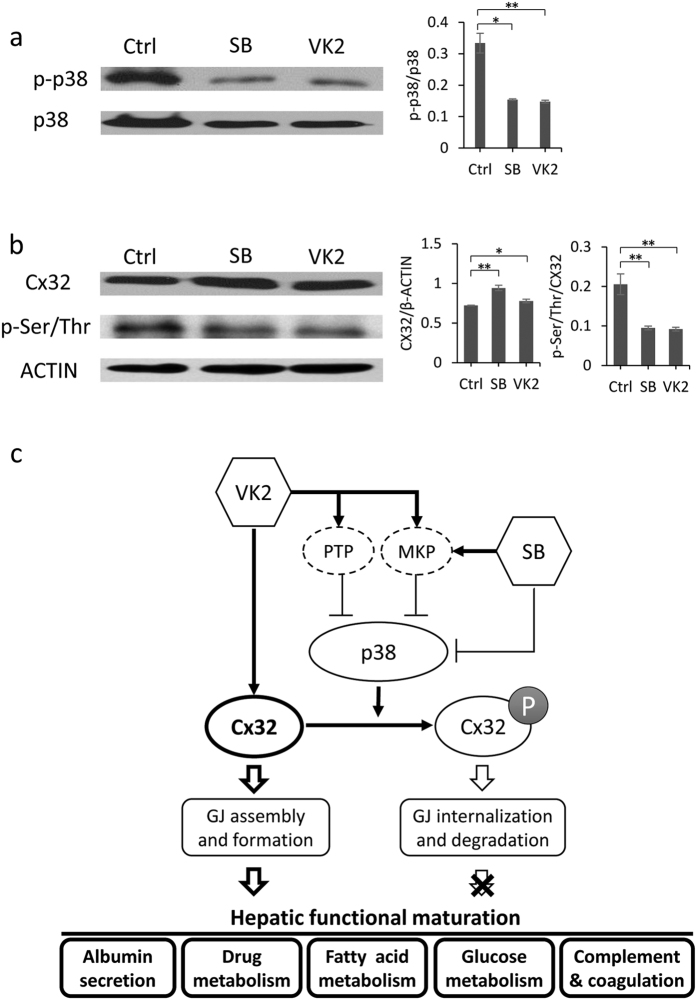
SB and VK2 modulate Cx32 phosphorylation. (**a**) Western blotting for phospho-p38 MAPK (p-p38) and total p38 MAPK (p38) of hESC-Heps induced with or without SB and VK2. (**b**) Cx32 immuno-precipitates from different cells were subjected to sodium dodecyl sulfate–polyacrylamide gel electrophoresis and Western blotting with anti-Cx32 and anti-phosphoserine/threonine. Protein loading was confirmed using anti–β-actin. (**c**) Model for the effect of SB and VK2 on hepatic differentiation. Apart from SB’s direct inhibition of p38 and VK2’s transcriptional activation of Cx32, SB and VK2 negatively regulated the p38 signaling pathway in similar ways, which in turn up-regulated Cx32 expression and drive hepatic functional maturation. Data represent mean ± SEM. *P < 0.05, **P < 0.01.

Cxs are dynamic polytopic membrane proteins that exhibit unprecedented short half-lives of only a few hours. MAP kinases, including p38, have been reported to phosphorylate Cxs and contribute to gap junction internalization and degradation^34^,^38^,^39^. Western blotting showed that phosph-p38 (p-p38) was dramatically down-regulated in hESC-Heps treated with SB, indicating efficient inhibition of p38 activity by SB (Fig. 8a). To investigate the effects of SB on Cx32 phosphorylation, we immune-precipitated Cx32 from untreated and SB–treated hESC-Heps and then probed the precipitates with anti-phosphoserine/threonine by Western blotting. SB-treatment reduced the levels of serine/threonine-phosphorylated Cx32 (Fig. 8b). Similarly, VK2-induced hepatocytes also showed much lower activity of p38 and less phosphorylated Cx32 than untreated cells (Fig. 8a,b). These results indicated that both SB and VK2 might stabilize Cx32 by inhibiting its phosphorylation, thus maintaining Cx32-mediated gap junctions (Fig. 8c).

## Discussion

Gap junctions form a web of cellular communication for the interconnected cells, which facilitate the spread of signals and cell-to-cell synchronization. Especially in liver, they play an essential role in amplifying injury, shown with the gap junction-dependent progression of liver injury induced by some drugs^37^. In cultured hepatocytes, sharing intracellular components via gap junction-mediated cell-to-cell communication resulted in synchronized death or alternatively led to the protection against toxic stimuli to death by an equal distribution of death versus survival molecules^40^. In these scenarios, Cx32 acted as one of the major members to compose gap junction structure. Until now, it has not been reported whether Cx32 and Cx32-mediated GJIC activities play any roles during the *in vitro* process of hepatic lineage restriction when pluripotent stem cells are guided to reach to a mature hepatocyte fate. Therefore, study on Cx32 expression as well as Cx-mediated GJIC activation on both hepatic differentiation from hESCs and maturation of hESC-Heps will be helpful to enlarge our knowledge on understanding Cx32 and the cell-cell interactions during liver organogenesis. Furthermore, it could be useful to improve the present *in vitro* induction strategies of hepatic differentiation and maturation from hESCs to more productive level.

Here we demonstrated the first evidence during process of hepatic differentiation from hESCs, which is that modulation of Cx32 as a key hepatic gap junction protein, to concisely regulate the hepatic lineage restriction and maturation of hESCs. Up-regulation of Cx32 expression and GJIC activity by the treatment of VK2 was shown to enhance the expression of hepatic markers and improve hepatic functions of hESC-Heps. On the other hand, inhibition of Cx32 by 2-APB blocked GJIC activity between cells, shown with the impeded hepatocyte maturation. These findings indicate that the establishment of natural and routine GJIC activity is critical for functional maturation of hESC-Heps.

GJIC activity is regulated by Cx biosynthesis, transport and assembly as well as the formation and removal of gap junctions from the cell surface. Indeed, modulation of Cx32 expression levels from our findings supported the positive function of Cx32 for hESC hepatic differentiation and maturation. On the other hand, a stably maintained up-regulation as well as homeostasis for GJIC is also important for this process. Phosphorylation of Cxs has been implicated in the regulation of gap junction turnover. All members of MAP kinase family, including p38, ERKs, and JNK, have been reported to phosphorylate Cxs, leading to gap junction internalization and eventually degradation^34^,^38^,^39^. Previous studies showed that p38 inhibitor, SB, blocked the down-regulation of Cx32 and well maintained gap junction structures *in vivo* after partial hepatectomy^35^. In this study, we demonstrated that the mechanism of p38-mediated phosphorylation for Cx32 degradation exists in the dynamically process from hESCs to differentiation into hepatocytes. When SB was applied to the stem cells during differentiation, p38 phosphorylation-dependent degradation of Cx32 can be successfully blocked and resulted in promoting hepatic differentiation of hESCs. In contrast, activation of p38 by the activator anisomycin blocked differentiation and maturation of hESC-Heps. Taken together, these observations supply the evidence that the mechanism of MAP kinase down-regulation on Cx also existed in the *in vitro* dynamic process from hESCs to hepatic cells. Significantly, that the lineage maturation of hESC-Heps could be modulated by restraint effect of p38 inhibitor (e.g. SB) on gap junction internalization, besides the direct modulation on up-regulation of Cx32 expression levels by VK2.

Bile acid secretion and drug metabolites modification via apical bile canaliculi are based on the critical function of hepatocytes’ epithelial polarization^41^,^42^. Gap junction plaques are frequently associated with tight junction strands in hepatocytes. Previous studies indicated that Cx32-mediated GJIC induced expression and function of tight junctions, which may affect cell polarity^43^,^44^,^45^. Our data indicated that the increased levels of expression and functions of Cx32 by SB or VK2 resulted in coordinated, functional polarization and bile canaliculi formation, whereas inhibition of Cx32 by 2-APB resulted in poorly polarized cells.

Moreover, for the first time, our results demonstrated that hESC-Heps carried the capacities to express the significantly increased levels of phase I and II enzymes, as well as phase III transporters, after the treatment with SB or VK2. In addition, they carried the significantly improved functional biotransforming systems, shown by HPLC analysis of drug metabolism. More importantly, the metabolic functions of hESC-Heps derived after SB- or VK2-treatments were enhanced by a common CYP inducer Rifampicin, with a significant increasing of some nuclear receptors, key mediators regulating drug-metabolizing enzymes and transporters^46^, which indicated those hESC-Heps developed a full biotransformation system. Remarkably, the gene expression profiles of hESC-Heps after SB- or VK2-treatments clustered closer to adult than to fetal hepatocytes. Overall, the hESC-Heps induced with SB- or VK2 showed improved maturation for hepatic characters, and these findings suggested that up-regulation of Cx32 may be very helpful for maturation of hepatic functions.

Our findings first proved the existence of regulation mechanism on GJIC during the *in vitro* dynamic process from hESCs to hepatocytes. Up-regulation of Cx32 as well as GJIC improved the hepatic differentiation from hESCs and following up hepatic maturation, which also raise the possibility that other members of Cxs and gap junction channels could be regulated for improving hepatic differentiation and maturation. Our modification on induction strategy could greatly benefit in guiding hepatic lineage restriction from pluripotent stem cells to hepatocytes, which presently faces the challenges to overcome that derived hepatic cells mostly have a fetal hepatocyte phenotype. In addition, our findings also supply new information to realize the developmental mechanism of liver organogenesis, which is communication via Cx32-mediated gap junctions may play essential for hepatic lineage restriction and maturation of fetal hepatocytes in embryonic livers.

## Materials and Methods

### Human Subjects

Fetal liver tissues were obtained from abortion in the Chinese PLA General Hospital (Beijing, China) with informed patient consent. All experiments were performed in accordance with relevant guidelines and regulations and all experimental protocols were approved by the academic committee of the Beijing Institute of Transfusion Medicine and the ethics committee of the PLA General Hospital.

### Cell Culture

The human embryonic stem cell (hESCs) lines, H9 and H1, were grown in feeder-free conditions in six-well Nunclon surface plates (Nunc) coated with Matrigel (BD Biosciences) and maintained in mTESR1 media (Stem Cell Technologies). Cells were passaged at a 1:3~4 ratio using dispase (Invitrogen). All Matrigel plates were coated with a 1:80 dilution in Advanced DMEM-F12 (Life Technologies) and incubated at room temperature for at least 1 hr before use.

### Generation of Hepatocytes from ESCs

Human ESCs were passaged with Accutase (Sigma) and plated at a density of 100,000 cells/cm^2^ in mTeSR with 10 μM Y27632 (Selleck) on Matrigel (BD), Laminin (BD), and collagen IV (BD) (3:1:1) mixed gel coated-plate (Corning). In the restriction of definitive endoderm (DEs) stage (S1), cells were cultured for 24 hrs in RPMI with B27 supplement (1:50, Gibco), 100 ng/ml Activin A (R&D) and 3 μM CHIR99021 (Selleck), and then treated with 100 ng/ml Activin A for 2 days. In the hepatic specification stage to get hepatic progenitor cells (S2), the culture medium was replaced with RPMI (Gibco) supplemented with B27 supplement (1:50), 20 ng/ml BMP4 and 10 ng/ml FGF2 for 5 days. And in the stage of hepatic maturation (S3), cells were cultured in Hepatocyte Culture Medium (HCM, Lonza) with 20 ng/ml HGF, 10 ng/ml OSM and 1 μM dexamethasone for 10–15 days. During stages 2 and 3, cells are fed every 48 hr. The final stage was also carried out with 10 μM SB203580 (Selleck), 50 μM Vitamin K2 (Sigma), 50 μM 2-APB (Tocris), or 0.5 μM Anisomycin (Selleck), and 0.1% dimethyl sulfoxide (DSMO) was used as control, as described in the text. Cells were photographed during differentiation using a Nikon phase contrast microscope (Nikon Microscopes).

### Isolation and Culture of Human Fetal Hepatocytes

Fetal liver tissues at 21 gestational weeks were obtained from abortion with informed patient consent. Fetal liver cells were obtained as previously described^47^. The fetal liver tissue was cut into 1–3 mm^3^ fragments for digestion in RPMI 1640 containing type IV collagenase and deoxyribonuclease (Sigma) at 37 °C for 15–20 min, and the cells were plated on Matrigel, Laminin, and collagen IV (3:1:1) mixed gel coated-plate in HCM (Lonza) supplemented with 10% fetal bovine serum. Twelve hours later, cells were washed with RPMI medium and subsequently cultured in HCM with 25 ng/ml HGF, 10 ng/ml OSM and 1 μM Dex in the presence or absence of SB203580 for 4 days before messenger RNA (mRNA) analysis.

### Fluorescence Recovery after Photobleaching (FRAP)

FRAP measurements were performed by LSM 510 META using modifications of methods previously described^48^. After the cells incubated with 1 μM Calcein-AM (Invitrogen) for 25 min in the confocal plates, a single cell was bleached for 25 s using a 488-nm laser beam. The selected cells were recorded for the pre- and post-bleach scans at low laser power, and recovery of fluorescence was recorded over 5 min at 15-s intervals. Two unbleached cells in the same visual field were selected as reference to subtract the loss of photobleaching during the acquisition process. Subsequent analysis of recorded frame sequences was done using LSM Image Examiner software (ZEISS). All FRAP measurements were performed on 15–30 cells per experiment and repeated at least three times.

## Functional Assays

### Albumin Secretion

hESC-Heps and PHHs isolated freshly from adult livers were cultured in HCM (Lonza) without phenol red and the supernatant was collected 24 hrs after medium change. The amount of albumin in the supernatant was determined by the human albumin ELISA kit (Bethyl Laboratory) according to the manufacturer’s instructions.

### Urea Synthesis

The amounts of urea in the culture media were measured after the cells were incubated with 20 mM ammonium chloride. Urea concentrations were determined by QuantiChrom Urea Assay Kit (BioAssay Systems) according to the manufacturer’s instructions.

### CYP Induction and Metabolism Assay

The cells were incubated with 100 μM testosterone, 50 μM dextromethorphan and 10 μM omeprazole (substrates for CYP3A4, 2D6 and 2C19, respectively for 3 hrs at 37 °C. The supernatants were collected for measurement of metabolized compounds by API4000 HPLC-MS/MS (Applied Biosystems). Total cell numbers were used to normalize the data. To evaluate CYP450 induction, differentiated cells were cultured with 25 μM Rifampicin for 3 days, with media changed every day. CYP3C4 activity was quantified using P450-GloTM CYP3A4 Luciferin-IPA kit (Promega) per manufacturer’s instruction. Total RNA was extracted to measure the induction of CYP enzymes responding to Rifampicin by qRT-PCR.

### Periodic Acid Schiff Staining

For glycogen detection, the hESCs-derived hepatocytes were stained by periodic acid-schiff (PAS, Sigma) following the manufacturer’s instructions. Briefly, differentiated cells were fixed using 4% paraformaldehyde in PBS for 15 min, then incubated with Periodic acid for 5 min, washed with distilled water, and incubated with freshly prepared Schiff’s solution for 15 min.

### Uptake of Low-Density Lipoprotein (LDL)

Cells were incubated with 10 μg/ml DiI-Ac-LDL (Biomedical Technologies) for 4 hrs at 37 °C. Then cells were washed with PBS and nuclei were stained with Hoechst 33342 (Invitrogen).

### Oil Red O Staining

To detect lipid, differentiated cells were fixed with 4% paraformaldehyde in PBS and incubated 30 min in a freshly prepared solution of 0.5% Oil red O (Cayman).

### Carboxydichlorofluroscein Diacetate Assay

To evaluate bile canaliculi function, hESCs-derived hepatocytes were incubated with 2 μM of 5 (and 6)-carboxy-2′,7′- dichlorofluorescein diacetate (CDFDA, Sigma) for 30 min. Cells were subsequently washed with ice-cold PBS and imaging was performed on a confocal microscope (PerkinElmer). 5(6)-carboxy-2′,7′-dichlorofluorescein (CDF)-positive canaliculi were normalized to cell number.

### Quantitative real-time PCR analyses

Total RNA was isolated using an RNeasy extraction kit. RNA was reverse transcribed using Superscript II reverse transcriptase (Invitrogen) according to the manufacturer’s instructions. Quantitative real-time PCR (qRT-PCR) was performed with SYBR Green real-time PCR master mix (TOYOBO) on a Bio-Rad iQ5 Real-Time PCR detection system (Bio-Rad). The data were analyzed using the delta-delta Ct method. The primers are listed in Supplementary Table S1.

### Small interfering RNA transfection

The hESCs-derived hepatic progenitor cells were transfected with Cx32 siRNA (siCx32) or the non-specific siRNA (NSsiRNA) (Sigma) using Lipofectamine 2000 (Invitrogen). Following transfection, cells were incubated at 37 °C in a CO_2_ incubator for 48 h before being harvested for the detecting the knockdown efficiency. And the cells were further differentiated to hepatocytes for evaluating the effect of Cx32 knockdown on hepatocyte differentiation.

### RNA sequencing

The mRNA was extracted using RNeasy Micro Kit (Qiagen) and followed by reversed transcription and amplification (REPLI-g WTA Single Cell Kit, Qiagen). RNA sequencing was performed on HiSeq X ten (Illumina). The low quality parts of raw reads were filtered. Reads were mapped to UCSC GRCh38 reference by Tophat version 2.0.11. Read counts of each gene were summarized using HTSeq version 0.6.1p1. DESeq2 package was employed to detect differentially expressed genes (fold change above 1 with adjusted p-values below 0.01). Original data were uploaded to the Gene Expression Omnibus database (accession number GSE76098).

### Immunofluorescent Staining

Cells were fixed with 4% paraformaldehyde for 20 min at room temperature and blocked with 10% goat or donkey serum for 1 h, followed by incubation with primary antibodies at 4 °C overnight. Labeled isotype-specific secondary antibodies were added and incubated 1 h at room temperature. Cells were counterstained with 4′,6-diamidino-2-phenylindole (DAPI) for visualization of cell nuclei and observed using a confocal microscopy (PerkinElmer) and Volocity Software (PerkinElmer). Antibodies used in this study were summarized in Supplementary Table S2.

## Flow cytometry

Single cell suspensions were obtained by dissociation with Accutase for 3–5 min. Cell surface antigen staining was performed in PBS at 4 °C. Intracellular staining was performed with the BD Cytofix/Cytoperm™ Kit (BD Biosciences) according to the manufacturer’s instructions. Briefly, cells were fixed and permeabilized with BD Cytofix/Cytoperm solution for 20 min at 4 °C. Intracellular antigen staining was performed in BD Perm/Wash solution.

The stained cells were analyzed or sorted with BD FACSCalibur (BD Biosciences), and the data was analyzed using the Flowjo software (TreeStar). The sources and concentrations of primary, secondary antibodies and isotype controls are listed in Supplementary Table S2.

### Immunoprecipitation and Western blotting

Cells were harvested in lysis buffer (50 mM Tris-HCl, pH 7.4, 0.25 mM sodium-deoxycholate, 150 mM NaCl, 2 mM EDTA, 0.1% sodium dodecyl sulfate, 1% Triton X-100) containing protease and phosphatase inhibitors (Roche). Lysates were sonicated for 30 s, maintained on ice for 30 min, and then spun at 15,000 rpm for 15 min at 4 °C. The supernatant was precleared with Protein G–Sepharose (GE Healthcare) and 100 μg total protein was incubated with 2 μg Cx32 antibody (Sigma) at 4 °C for 2 hrs with rotation. Thereafter, 100 μl of Sepharose beads was added, and the sample was rotated at 4 °C for 4 hrs. The beads were collected and washed thoroughly in lysis buffer, and the precipitated proteins eluted with loading buffer (2 × Laemmli buffer plus dithiothreitol). Proteins were separated by 12% sodium dodecyl sulfate–polyacrylamide gel electrophoresis, transferred to polyvinylidene difluoride membranes, and probed with anti-Cx32, antiphosphoserine/threonine (Abcam), and horseradish-peroxidase-conjugated anti-rabbit or anti-mouse IgG antibodies. Proteins were detected by enhanced chemiluminescence HRP substrate (Millipore).

### Statistics

Data are shown as means and standard deviations. For most statistic evaluation, 2-tailed Student’s t test was applied for calculating statistical probability in this study. *P* values less than 0.05 were considered to be statistically significant. For fluorescence recovery analysis, the two-way ANOVA test was applied. For all statistics, data from at least three independent samples or repeated experiments were used.

## Additional Information

**How to cite this article**: Qin, J. *et al.* Connexin 32-mediated cell-cell communication is essential for hepatic differentiation from human embryonic stem cells. *Sci. Rep.* **6**, 37388; doi: 10.1038/srep37388 (2016).

**Publisher’s note:** Springer Nature remains neutral with regard to jurisdictional claims in published maps and institutional affiliations.

## Supplementary Material

Supplementary Information

## References

[b1] Dhawan A., Puppi J., Hughes R. D. & Mitry R. R. Human hepatocyte transplantation: current experience and future challenges. Nature reviews. Gastroenterology & hepatology 7, 288–298, doi: 10.1038/nrgastro.2010.44 (2010). 20368738

[b2] Gomez-Lechon M. J., Donato M. T., Castell J. V. & Jover R. Human hepatocytes in primary culture: the choice to investigate drug metabolism in man. Current drug metabolism 5, 443–462 (2004). 15544436 10.2174/1389200043335414

[b3] Guillouzo A. Liver cell models in *in vitro* toxicology. Environ Health Perspect 106 Suppl 2, 511–532 (1998). 9599700 10.1289/ehp.98106511 PMC1533385

[b4] Hewitt N. J. *et al.* Primary hepatocytes: current understanding of the regulation of metabolic enzymes and transporter proteins, and pharmaceutical practice for the use of hepatocytes in metabolism, enzyme induction, transporter, clearance, and hepatotoxicity studies. Drug Metab Rev 39, 159–234, doi: 10.1080/03602530601093489 (2007). 17364884

[b5] Touboul T. *et al.* Generation of functional hepatocytes from human embryonic stem cells under chemically defined conditions that recapitulate liver development. Hepatology (Baltimore, Md.) 51, 1754–1765, doi: 10.1002/hep.23506 (2010). 20301097

[b6] Si-Tayeb K. *et al.* Highly efficient generation of human hepatocyte-like cells from induced pluripotent stem cells. Hepatology (Baltimore, Md.) 51, 297–305, doi: 10.1002/hep.23354 (2010). PMC2946078 19998274

[b7] Basma H. *et al.* Differentiation and transplantation of human embryonic stem cell-derived hepatocytes. Gastroenterology 136, 990–999, doi: 10.1053/j.gastro.2008.10.047 (2009). 19026649 PMC2732349

[b8] Ma X. *et al.* Highly efficient differentiation of functional hepatocytes from human induced pluripotent stem cells. Stem cells translational medicine 2, 409–419, doi: 10.5966/sctm.2012-0160 (2013). 23681950 PMC3673753

[b9] Zhao D. *et al.* Promotion of the efficient metabolic maturation of human pluripotent stem cell-derived hepatocytes by correcting specification defects. Cell research 23, 157–161, doi: 10.1038/cr.2012.144 (2013). 23070301 PMC3541654

[b10] Avior Y. *et al.* Microbial-derived lithocholic acid and vitamin K drive the metabolic maturation of pluripotent stem cells-derived and fetal hepatocytes. Hepatology (Baltimore, Md.), doi: 10.1002/hep.27803 (2015). 25808545

[b11] Duan Y. *et al.* Differentiation and Enrichment of Hepatocyte-Like Cells from Human Embryonic Stem Cells *in vitro* and *in vivo*. Stem Cells 25, 3058–3068, doi: 10.1634/stemcells.2007-0291 (2007). 17885076

[b12] Baxter M. *et al.* Phenotypic and functional analyses show stem cell-derived hepatocyte-like cells better mimic fetal rather than adult hepatocytes. Journal of hepatology 62, 581–589, doi: 10.1016/j.jhep.2014.10.016 (2015). 25457200 PMC4334496

[b13] Saez J. C., Connor J. A., Spray D. C. & Bennett M. V. Hepatocyte gap junctions are permeable to the second messenger, inositol 1,4,5-trisphosphate, and to calcium ions. Proceedings of the National Academy of Sciences of the United States of America 86, 2708–2712 (1989). 2784857 10.1073/pnas.86.8.2708 PMC286987

[b14] Vinken M. Role of connexin-related signalling in hepatic homeostasis and its relevance for liver-based *in vitro* modelling. World journal of gastrointestinal pathophysiology 2, 82–87, doi: 10.4291/wjgp.v2.i5.82 (2011). 22013553 PMC3196623

[b15] Maes M., Crespo Yanguas S., Willebrords J., Cogliati B. & Vinken M. Connexin and pannexin signaling in gastrointestinal and liver disease. Translational research: the journal of laboratory and clinical medicine 166, 332–343, doi: 10.1016/j.trsl.2015.05.005 (2015). 26051630 PMC4570182

[b16] Zhao B. *et al.* Connexin32 regulates hepatoma cell metastasis and proliferation via the p53 and Akt pathways. Oncotarget 6, 10116–10133 (2015). 25426556 10.18632/oncotarget.2687 PMC4496344

[b17] Vinken M. Gap junctions and non-neoplastic liver disease. Journal of hepatology 57, 655–662, doi: 10.1016/j.jhep.2012.02.036 (2012). 22609308

[b18] Schiller P. C., D’Ippolito G., Brambilla R., Roos B. A. & Howard G. A. Inhibition of gap-junctional communication induces the trans-differentiation of osteoblasts to an adipocytic phenotype *in vitro*. The Journal of biological chemistry 276, 14133–14138, doi: 10.1074/jbc.M011055200 (2001). 11278824

[b19] Li Z., Zhou Z., Saunders M. M. & Donahue H. J. Modulation of connexin43 alters expression of osteoblastic differentiation markers. American journal of physiology. Cell physiology 290, C1248–C1255, doi: 10.1152/ajpcell.00428.2005 (2006). 16319124

[b20] Long A. C., Bomser J. A., Grzybowski D. M. & Chandler H. L. All-trans retinoic Acid regulates cx43 expression, gap junction communication and differentiation in primary lens epithelial cells. Current eye research 35, 670–679, doi: 10.3109/02713681003770746 (2010). 20673043

[b21] Li S. *et al.* Connexin43-containing gap junctions potentiate extracellular Ca(2+)-induced odontoblastic differentiation of human dental pulp stem cells via Erk1/2. Experimental cell research 338, 1–9, doi: 10.1016/j.yexcr.2015.09.008 (2015). 26376117

[b22] Neveu M. J. *et al.* Proliferation-associated differences in the spatial and temporal expression of gap junction genes in rat liver. Hepatology (Baltimore, Md.) 22, 202–212 (1995). 7601414

[b23] Neveu M. J. *et al.* Colocalized alterations in connexin32 and cytochrome P450IIB1/2 by phenobarbital and related liver tumor promoters. Cancer research 54, 3145–3152 (1994). 8205533

[b24] Shoda T. *et al.* The relationship between decrease in Cx32 and induction of P450 isozymes in the early phase of clofibrate hepatocarcinogenesis in the rat. Archives of toxicology 73, 373–380 (1999). 10550479 10.1007/s002040050676

[b25] Shoda T. *et al.* Liver tumor-promoting effect of beta-naphthoflavone, a strong CYP 1A1/2 inducer, and the relationship between CYP 1A1/2 induction and Cx32 decrease in its hepatocarcinogenesis in the rat. Toxicologic pathology 28, 540–547 (2000). 10930040 10.1177/019262330002800406

[b26] Nelles E. *et al.* Defective propagation of signals generated by sympathetic nerve stimulation in the liver of connexin32-deficient mice. Proceedings of the National Academy of Sciences of the United States of America 93, 9565–9570 (1996). 8790370 10.1073/pnas.93.18.9565 PMC38468

[b27] Stumpel F., Ott T., Willecke K. & Jungermann K. Connexin 32 gap junctions enhance stimulation of glucose output by glucagon and noradrenaline in mouse liver. Hepatology (Baltimore, Md.) 28, 1616–1620, doi: 10.1002/hep.510280622 (1998). 9828226

[b28] Yang J., Ichikawa A. & Tsuchiya T. A novel function of connexin 32: marked enhancement of liver function in a hepatoma cell line. Biochemical and Biophysical Research Communications 307, 80–85, doi: 10.1016/s0006-291x(03)01117-3 (2003). 12849984

[b29] Temme A. *et al.* Dilated bile canaliculi and attenuated decrease of nerve-dependent bile secretion in connexin32-deficient mouse liver. Pflugers Archiv: European journal of physiology 442, 961–966 (2001). 11680630 10.1007/s004240100623

[b30] Paku S., Nagy P., Kopper L. & Thorgeirsson S. S. 2-acetylaminofluorene dose-dependent differentiation of rat oval cells into hepatocytes: confocal and electron microscopic studies. Hepatology (Baltimore, Md.) 39, 1353–1361, doi: 10.1002/hep.20178 (2004). 15122764

[b31] Zhang M. & Thorgeirsson S. S. Modulation of connexins during differentiation of oval cells into hepatocytes. Experimental cell research 213, 37–42, doi: 10.1006/excr.1994.1170 (1994). 7517369

[b32] Rosenberg E. *et al.* Correlation of expression of connexin mRNA isoforms with degree of cellular differentiation. Cell adhesion and communication 4, 223–235 (1996). 9117343 10.3109/15419069609010768

[b33] Kaneda M. *et al.* Vitamin K2 suppresses malignancy of HuH7 hepatoma cells via inhibition of connexin 43. Cancer letters 263, 53–60, doi: 10.1016/j.canlet.2007.12.019 (2008). 18249064

[b34] Polontchouk L., Ebelt B., Jackels M. & Dhein S. Chronic effects of endothelin 1 and angiotensin II on gap junctions and intercellular communication in cardiac cells. FASEB journal: official publication of the Federation of American Societies for Experimental Biology 16, 87–89, doi: 10.1096/fj.01-0381fje (2002). 11709493

[b35] Yamamoto T. *et al.* p38 MAP-kinase regulates function of gap and tight junctions during regeneration of rat hepatocytes. Journal of hepatology 42, 707–718, doi: 10.1016/j.jhep.2004.12.033 (2005). 15826721

[b36] Tao L. & Harris A. L. 2-aminoethoxydiphenyl borate directly inhibits channels composed of connexin26 and/or connexin32. Molecular pharmacology 71, 570–579, doi: 10.1124/mol.106.027508 (2007). 17095584

[b37] Patel S. J. *et al.* Gap junction inhibition prevents drug-induced liver toxicity and fulminant hepatic failure. Nature biotechnology 30, 179–183, doi: 10.1038/nbt.2089 (2012). PMC3609650 22252509

[b38] Petrich B. G. *et al.* c-Jun N-Terminal Kinase Activation Mediates Downregulation of Connexin43 in Cardiomyocytes. Circulation research 91, 640–647, doi: 10.1161/01.res.0000035854.11082.01 (2002). 12364393

[b39] Thevenin A. F. *et al.* Proteins and mechanisms regulating gap-junction assembly, internalization, and degradation. Physiology (Bethesda) 28, 93–116, doi: 10.1152/physiol.00038.2012 (2013). 23455769 PMC3768091

[b40] Saito C., Shinzawa K. & Tsujimoto Y. Synchronized necrotic death of attached hepatocytes mediated via gap junctions. Scientific reports 4, 5169, doi: 10.1038/srep05169 (2014). 24893927 PMC4044626

[b41] Khetani S. R. & Bhatia S. N. Microscale culture of human liver cells for drug development. Nature Biotechnology 26, 120–126, doi: 10.1038/nbt1361 (2007). 18026090

[b42] Kidambi S. *et al.* Oxygen-mediated enhancement of primary hepatocyte metabolism, functional polarization, gene expression, and drug clearance. Proceedings of the National Academy of Sciences of the United States of America 106, 15714–15719, doi: 10.1073/pnas.0906820106 (2009). 19720996 PMC2747185

[b43] Kojima T. *et al.* Cx32 but not Cx26 is associated with tight junctions in primary cultures of rat hepatocytes. Experimental cell research 263, 193–201, doi: 10.1006/excr.2000.5103 (2001). 11161718

[b44] Kojima T. *et al.* Cx32 formation and/or Cx32-mediated intercellular communication induces expression and function of tight junctions in hepatocytic cell line. Experimental cell research 276, 40–51, doi: 10.1006/excr.2002.5511 (2002). 11978007

[b45] Kojima T., Murata M., Go M., Spray D. C. & Sawada N. Connexins induce and maintain tight junctions in epithelial cells. The Journal of membrane biology 217, 13–19, doi: 10.1007/s00232-007-9021-4 (2007). 17568974

[b46] Hewitt N. J., Lecluyse E. L. & Ferguson S. S. Induction of hepatic cytochrome P450 enzymes: methods, mechanisms, recommendations, and *in vitro*-*in vivo* correlations. Xenobiotica; the fate of foreign compounds in biological systems 37, 1196–1224, doi: 10.1080/00498250701534893 (2007). 17968743

[b47] Lilja H. *et al.* Fetal rat hepatocytes: isolation, characterization, and transplantation in the Nagase analbuminemic rats. Transplantation 64, 1240–1248 (1997). 9371663 10.1097/00007890-199711150-00003

[b48] Wade M. H., Trosko J. E. & Schindler M. A fluorescence photobleaching assay of gap junction-mediated communication between human cells. Science (New York, N.Y.) 232, 525–528 (1986). 10.1126/science.3961495 3961495

