# Author Correction: Connexin 32-mediated cell-cell communication is essential for hepatic differentiation from human embryonic stem cells

**DOI:** 10.1038/s41598-020-62384-2

**Published:** 2020-04-06

**Authors:** Jinhua Qin, Mingyang Chang, Shuyong Wang, Zhenbo Liu, Wei Zhu, Yi Wang, Fang Yan, Jian Li, Bowen Zhang, Guifang Dou, Jiang Liu, Xuetao Pei, Yunfang Wang

**Affiliations:** 1 Stem Cell and Regenerative Medicine Lab, Beijing Institute of Transfusion Medicine, Beijing, 100850 China; 2 Tissue Engineering Lab, Beijing Institute of Transfusion Medicine, Beijing, 100850 China; 3 South China Research Center for Stem Cell and Regenerative Medicine, South China Institute of Biomedicine, Guangzhou, 510005 China; 4 CAS Key Laboratory of Genome Sciences and Information, Beijing Institute of Genomics, Chinese Academy of Sciences, Beijing, 100101 China; 5 Laboratory of Hematological Pharmacology, Beijing Institute of Transfusion Medicine, Beijing, 100850 China

Correction to: *Scientific Reports* 10.1038/srep37388, published online 22 November 2016

This Article contains errors in Figure 5c. The panel for Dil-ac-LDL and panel for Oil red O in the VK2-treated group were prepared incorrectly due to misfiling of data. In addition, there are errors in the length of the scale bars.

As a result, the legend for Figure 5c,

“(**c**) Dil-ac-LDL uptake, oil red O staining, PAS staining, and CDFDA staining of hESC-Heps induced with SB, VK2 or 2-APB. Staining intensity was normalized to cell number. Scale bars, 50 μm. Data represent mean ± SEM. **P < 0.01, ***P < 0.001.”

should read:

“(**c**) Dil-ac-LDL uptake, oil red O staining, PAS staining, and CDFDA staining of hESC-Heps induced with SB, VK2 or 2-APB. Staining intensity was normalized to cell number. Scale bars, 100 μm. Data represent mean ± SEM. **P < 0.01, ***P < 0.001.”

The correct Figure 5c and accompanying figure legend appear below as Figure 1.

**Figure 1:**
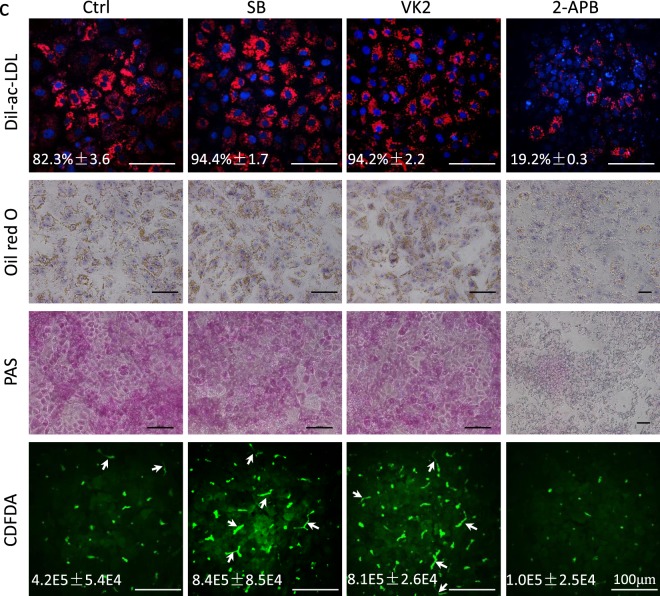
(**c**) Dil-ac-LDL uptake, oil red O staining, PAS staining, and CDFDA staining of hESC-Heps induced with SB, VK2 or 2-APB. Staining intensity was normalized to cell number. Scale bars, 100 μm. Data represent mean ± SEM. **P < 0.01, ***P < 0.001.

