# Supplementary material for: Connexin 32-mediated cell-cell communication is essential for hepatic differentiation from human embryonic stem cells
Source: Sci Rep. 2016 Nov 22;6:37388. doi: 10.1038/srep37388 (PMC5118817; doi:10.1038/srep37388)
Supplement: Supplementary Information [file srep37388-s1.pdf]

Supplementary information

Connexin 32-mediated cell-cell communication is essential for hepatic differentiation from human embryonic stem cells

Jinhua Qin[1,2,3], Shuyong Wang[1,2,3], Zhenbo Liu[4], Wei Zhu[4], Yi Wang[2], Fang Yan[2], Jian Li[5], Bowen Zhang[1,3], Mingyang Chang[2], Guifang Dou[5], Jiang Liu[4], Xuetao Pei[1,3,*] & Yunfang Wang[2,*]

[1] Stem cell and Regenerative Medicine Lab, [2] Tissue Engineering Lab, and [5]Laboratory of Hematological Pharmacology, Beijing Institute of Transfusion Medicine, Beijing 100850, China.

[3] South China Research Center for Stem Cell & Regenerative Medicine, AMMS, Guangzhou 510005, China.

[4] CAS Key Laboratory of Genome Sciences and Information, Beijing Institute of Genomics, Chinese Academy of Sciences, Beijing 100101, China.

[*] Correspondence: peixt@nic.bmi.ac.cn (X. P.); wangyf1972@gmail.com (Y. W.)

Supplementary Figure 1 (related to **Fig. 1**)

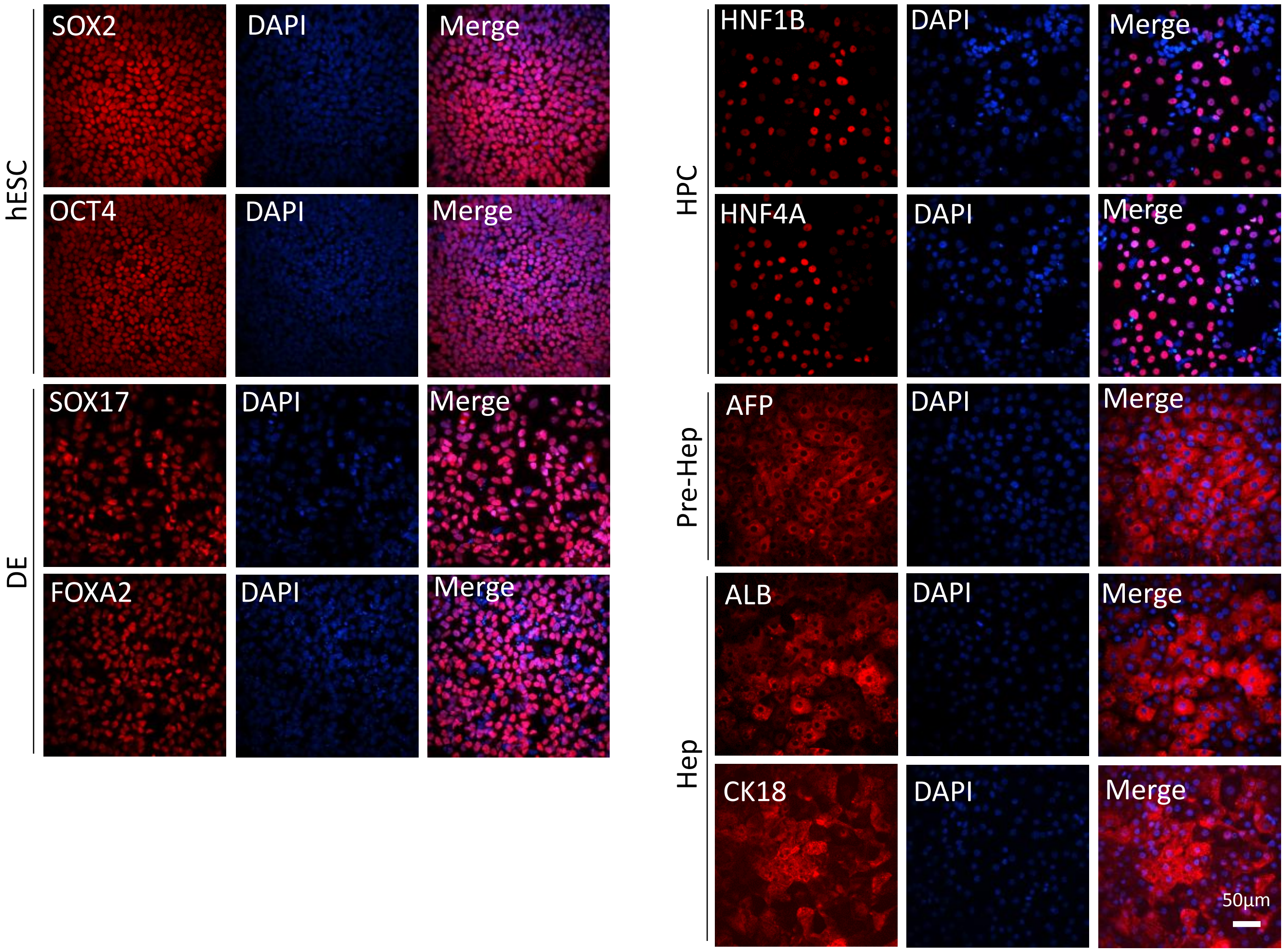

Supplementary Figure 1. Hepatocyte differentiation from hESCs was monitored by immunocytochemistry at different stages using antibodies that recognized SOX2, OCT4, FOXA2, SOX17, HNF1B, HNF4A, AFP, ALB and CK18. hESC, human embryonic stem cell; DE, definitive endoderm; HPC, hepatic progenitor cell; Pre-Hep, the early stage of hepatocyte-like cells at about day 15; Hep, hepatocytes derived from hESCs. Scale bars, 50 μm.

Supplementary Figure 2 (related to **Fig. 2**)

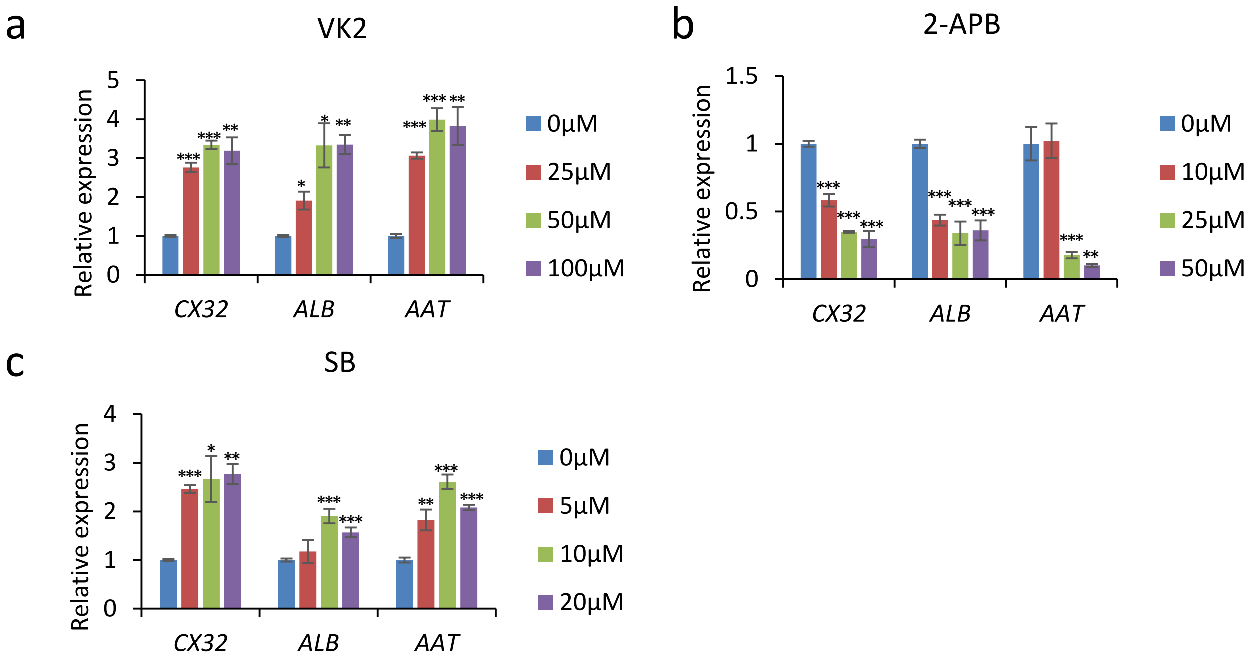

Supplementary Figure 2. qRT-PCR analysis of hESC-Heps exposed to increasing concentrations of VK2 (a), 2-APB (b) or SB (c) during the final maturation stage. VK2 and SB caused a dose-dependent induction of Cx32, ALB and AAT expression, while addition of 2-APB to the last stage of differentiation caused reduction of these genes. Data represent mean ± SEM. *P < 0.05, **P < 0.01, ***P < 0.001.

Supplementary Figure 3 (related to **Fig. 1c and 2b**)

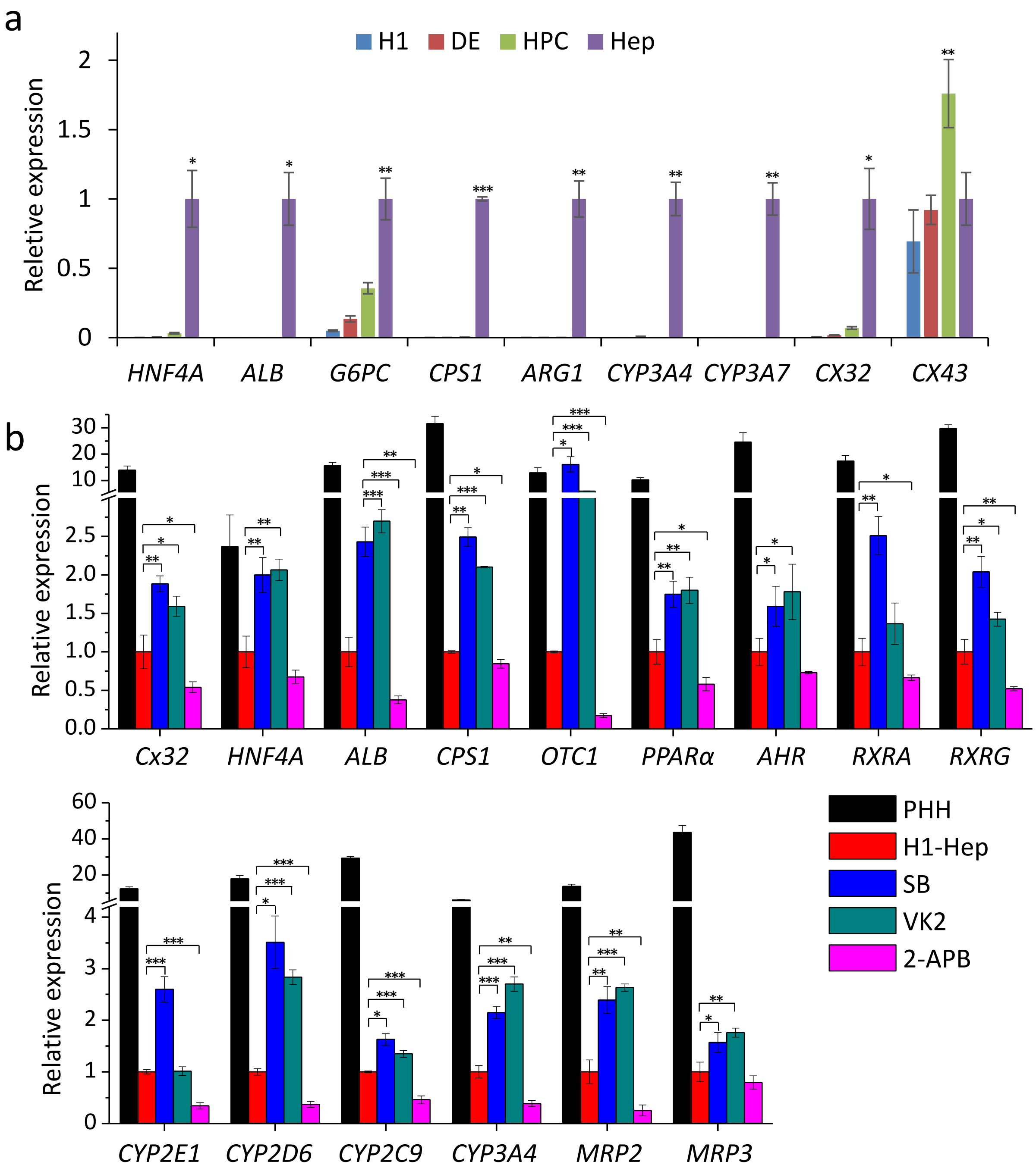

Supplementary Figure 3. H1 hESCs can be also induced into hepatocytes. (a) qRT-PCR analysis of key stages of H1 hESCs differentiation to hepatocytes. H1, H1 hESC line; DE, definitive endoderm; HPC, hepatic progenitor cell; Hep, hepatocytes derived from hESCs. (b) qRT-PCR analysis of hepatocytes derived from H1 hESCs (H1-Hep) induced with SB, VK2 or 2-APB. Data represent mean ± SEM. $*P < 0.05$, $**P < 0.01$, $***P < 0.001$.

Supplementary Figure 4 (related to **Fig. 2c** and **3c**)

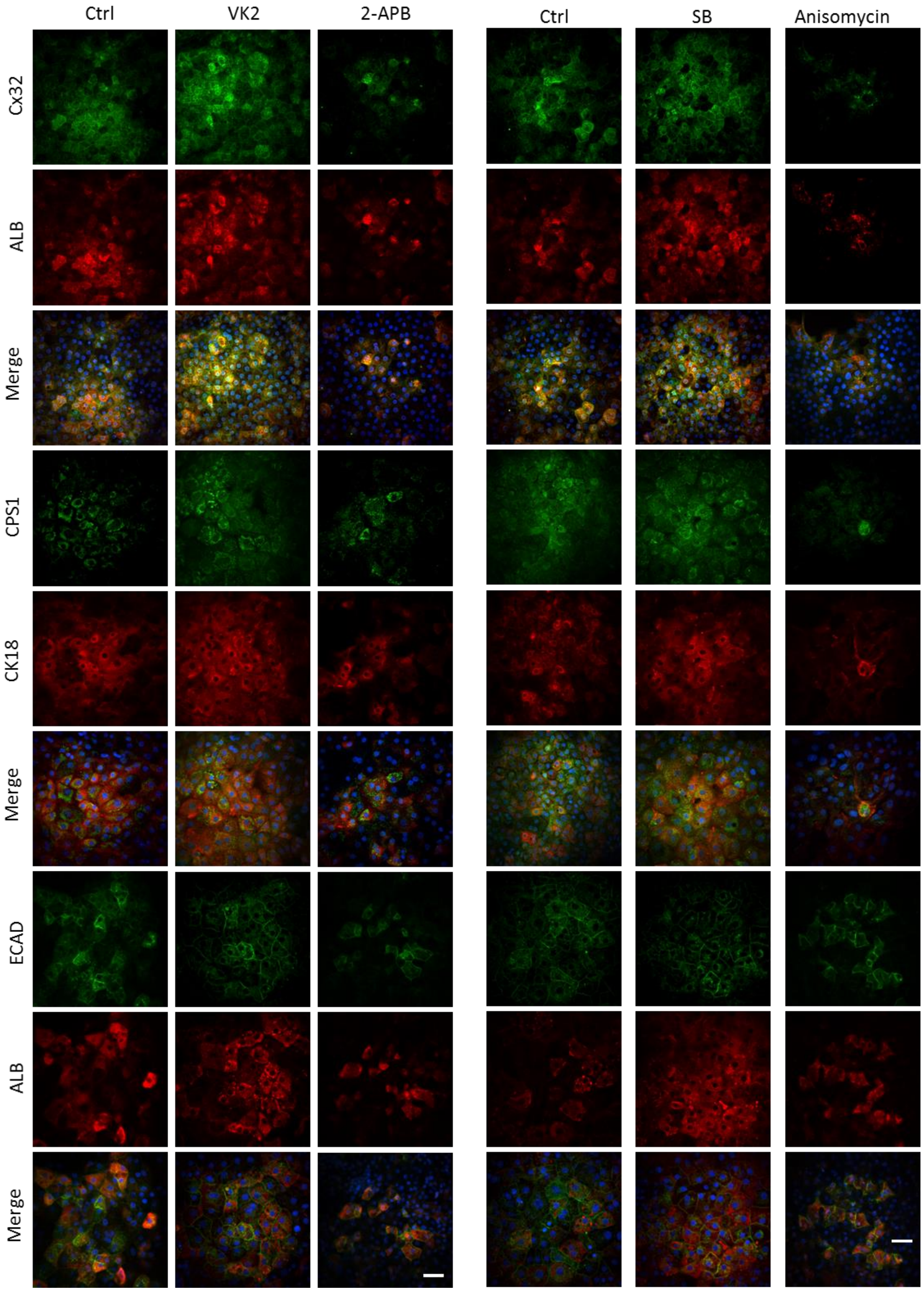

Supplementary Figure 4. Immunostaining of Cx32, ALB, CPS1, CK18 and ECAD in hESC-Heps induced with VK2, 2-APB, SB or Anisomycin. Scale bars, 50 μm.

Supplementary Figure 5

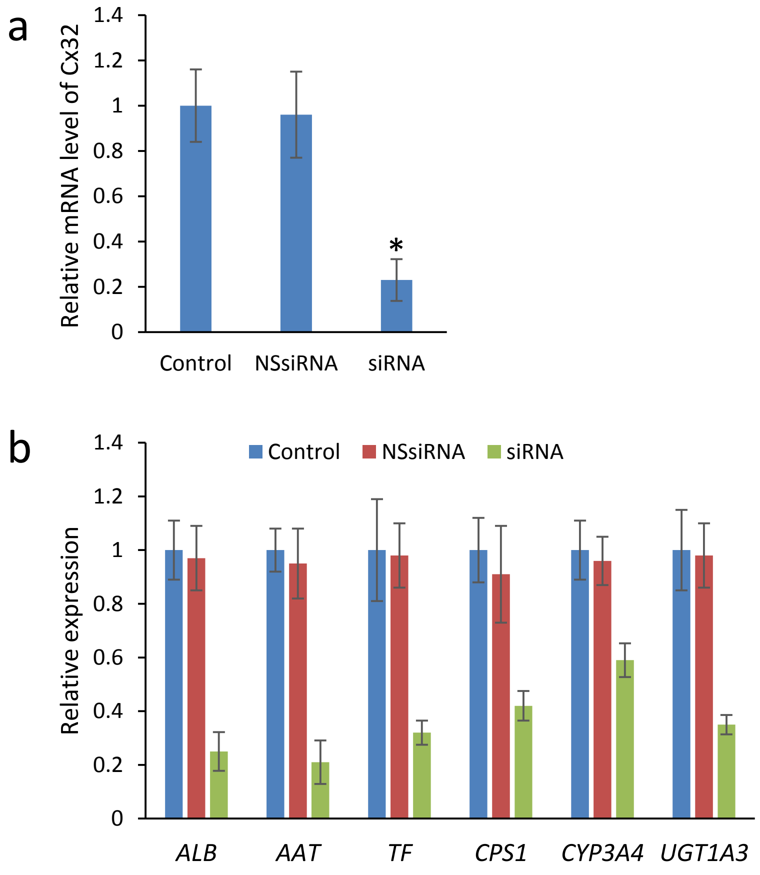

Supplementary Figure 5. Knockdown of Cx32 in hESCs-derived hepatic progenitor cells resulted in impaired hepatocyte differentiation. (a) Cx32 mRNA expression was detected by qRT-PCR at 48 h after transfected with Cx32 siRNA and non-specific siRNA (NSsiRNA). (b) qRT-PCR analysis of hepatocyte markers in cells differentiated from hESCs-derived hepatic progenitor cells with or without Cx32 knockdown. Data represent mean ± SEM. (*$P < 0.05$ vs. control)

Supplementary Figure 6 (related to **Figs. 1c, d and 2b**)

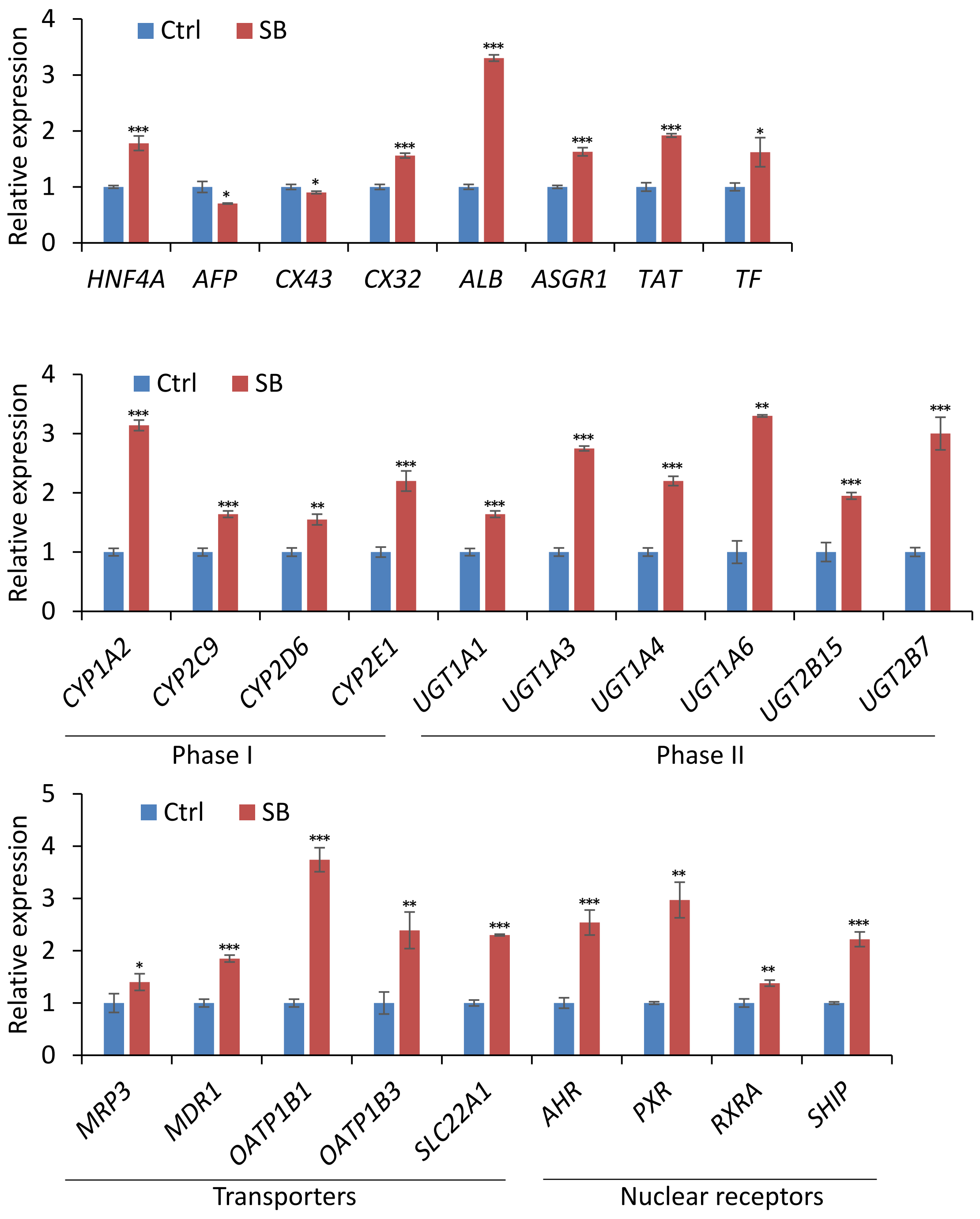

Supplementary Figure 6. SB drove fetal human hepatocyte maturation. qRT-PCR analysis of fetal human hepatocytes cultured for 4 days with SB or in its absence (ctrl). Data represent mean ± SEM. *$P < 0.05$, **$P < 0.01$, ***$P < 0.001$.

Supplementary Figure 7 (related to **Fig. 8**)

a

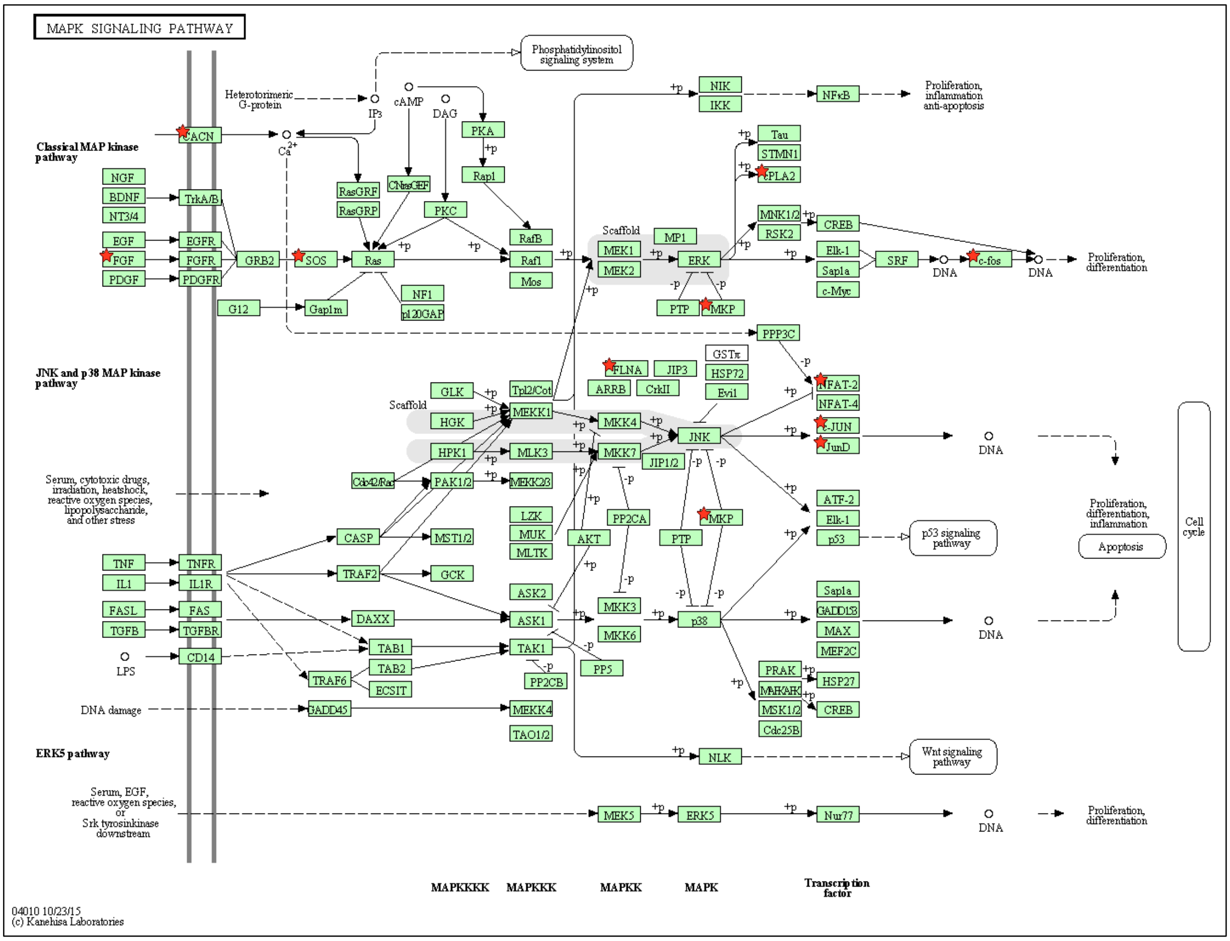

b

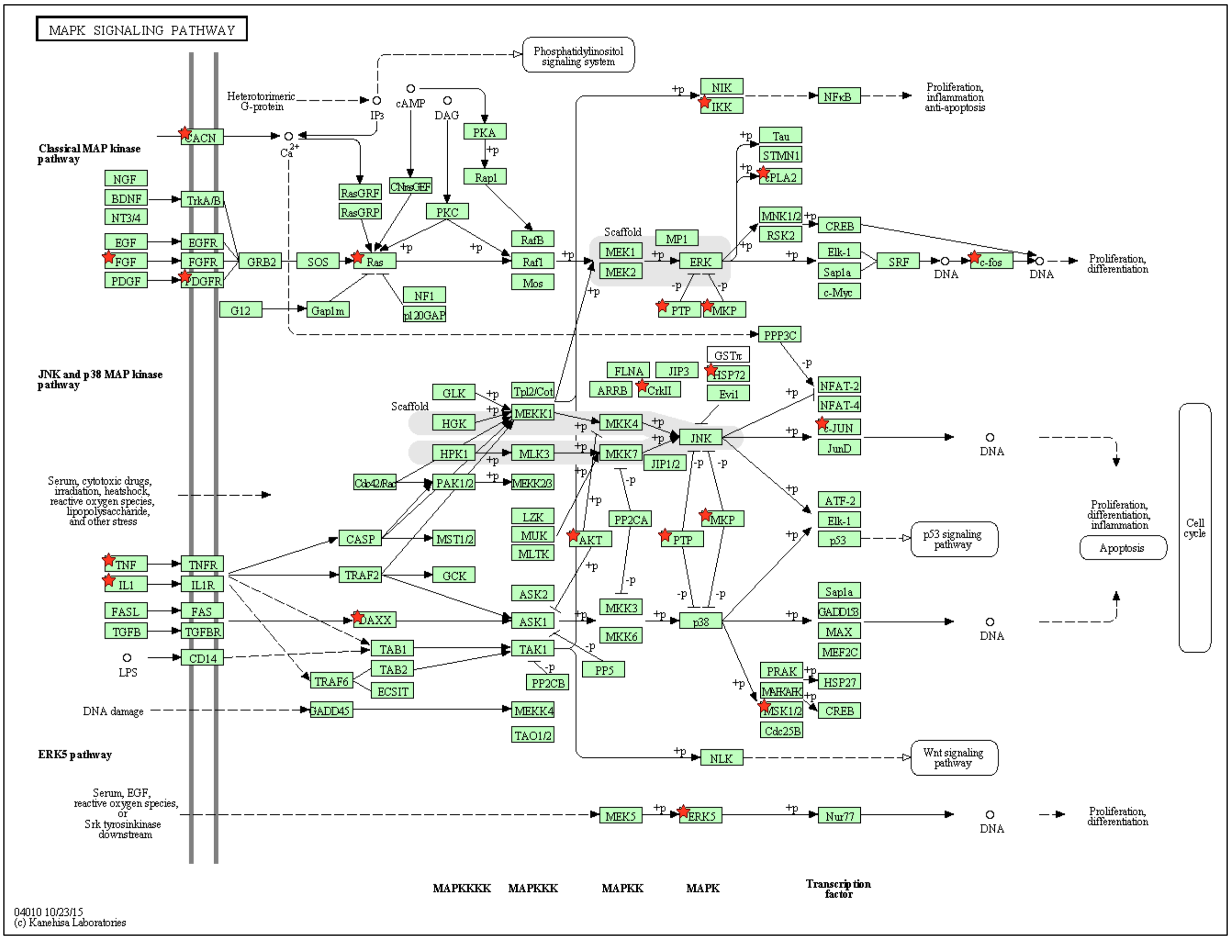

Supplementary Figure 7. Mapping of genes upregulated in SB (a) or VK2 (b)-induced hESC-Heps in MAPK signaling pathway, compared to untreated control. The map of MAPK signaling pathway is obtained by KEGG[1,2].

Supplementary Figure 8 (related to **Fig. 8**)

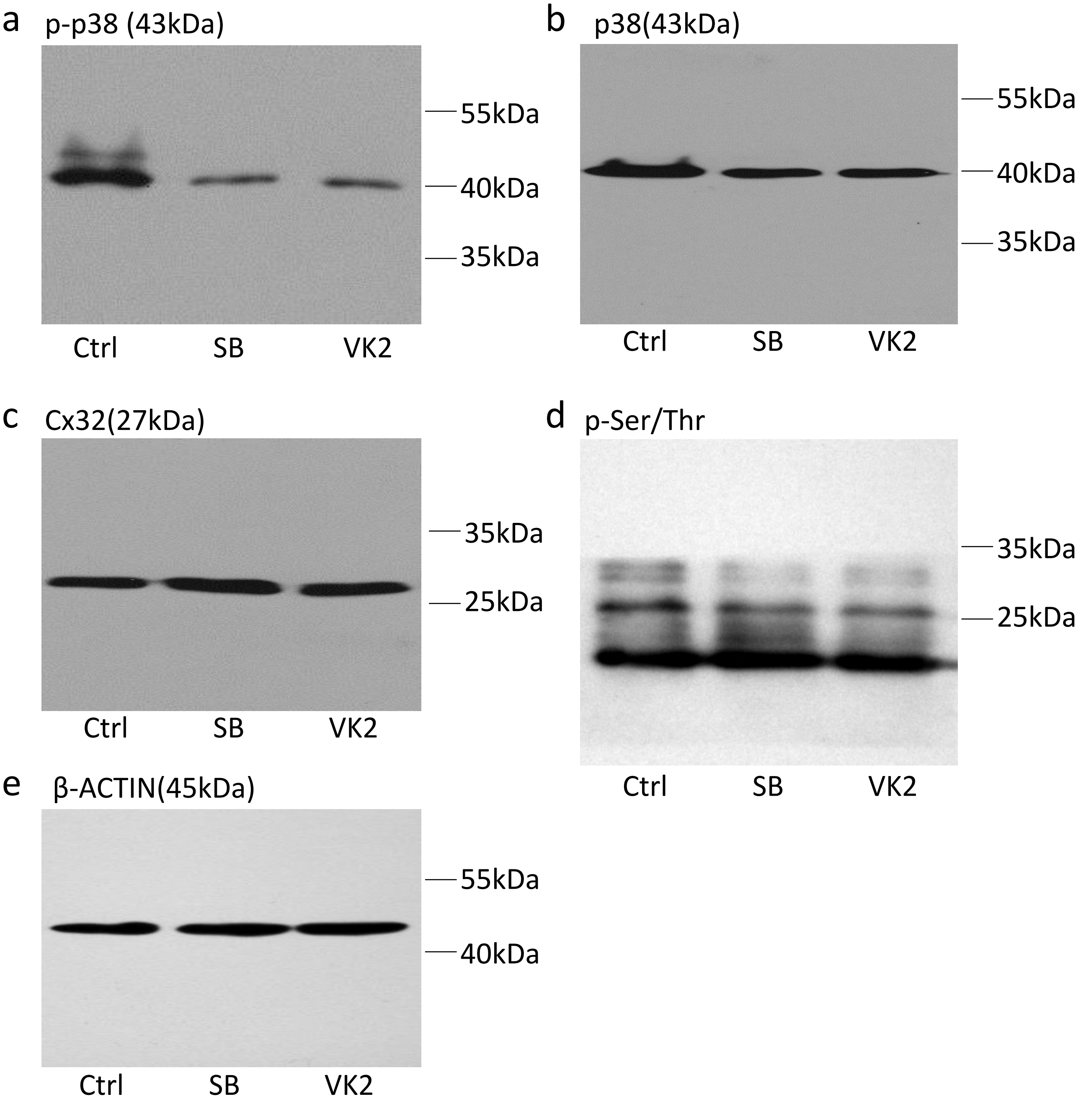

Supplementary Figure 8. Full length images (reported in Fig. 8) from a single Western blot membrane probed for (a) phospho-p38 MAPK (p-p38) , (b) total p38 MAPK (p38), (c) Cx32, (d) anti-phosphoserine/threonine (p-Ser/Thr) and (e) β-ACTIN as a load control.

Supplementary Table 1. Primers used in this study.

Gene	Forward	Reward
GAPDH	GAGTCAACGGATTTGGTCGT	TTGATTTTGGAGGGATCTCG
OCT4	CTTGAATCCCGAATGGAAAGGG	GTGTATATCCCAGGGTGATCCTC
C-MYC	TCGGAAGGACTATCCTGCTG	GTGTGTTCGCCTCTTGACATT
NANOG	ACAACTGGCCGAAGAATAGCA	GGTTCCCAGTCGGGTTCAC
SOX17	GTGGACCGCACGGAATTTG	GGAGATTCACACCGGAGTCA
FOXA2	GCGACCCCAAGACCTACAG	GGTTCTGCCGGTAGAAGGG
GATA4	CCCAGACGTTCTCAGTCAGTG	GCTGTTCCAAGAGTCCTGCT
HNF4A	ACGGACAGATGTGTGAGTGG	CAGGAGCTTATAGGGCTCAGA
HNF1B	TGTACGCACACAAGCAGGAA	GTTGGTGAGTGTACTGATGCTG
AFP	CTTGCACACAAAAAGCCCACT	GGGATGCCTTCTTGCTATCTCAT
ALB	TTTATGCCCCGGAACTCCTTT	ACAGGCAGGCAGCTTTATCAG
TF	CCTCCTACCTTGATTGCATCAG	TTTTGACCCATAGAACTCTGCC
AAT	ATGCTGCCCAGAAGACAGATA	TTGTTGAAGGTTGGGTGATCC
CX32	TTCCCCATCTCCCATGTGC	CCCTCAAGCCGTAGCATTTTC
CX43	GTCTGCTATGACAAGTCTTTCCC	AGAACACATGAGCCAGGTACA
CLDN	CCCTTTTAGGAGGTAGTGTAGGC	CCGTAGCCATAGCCATAACCA
CPS1	GGCCATCCATCCTCTGTTGC	GCTAAGTCCCAGTTCATCCA
OTC1	TCGAGCCAATACTGCATCTG	CTTCTGGGAGGACATCCTTG
ARG1	TGGACAGACTAGGAATTGGCA	CCAGTCCGTCAACATCAAAACT
G6PC	TCAGGGAAAGATAAAGCCGACC	AGGTAGATTCGTGACAGACAGAC
CYP1A2	ATGGCATTGTCCCAGTCTGTT	TGGCTCTGGTGGACTTTTCAG
CYP3A4	AAGTCGCCTCGAAGATACACA	AAGGAGAGAACACTGCTCGTG
CYP3A7	AAGGTCGCCTCAAAGAGACA	TGCACTTTCTGCTGGACATC
CYP2C9	GCCACATGCCCTACACAGATG	TAATGTCACAGGTCACTGCATGG
CYP2D6	GTGTCCAACAGGAGATCGACG	CACCTCATGAATCACGGCAGT
CYP2E1	CTGACCACCCTCCGGAACTA	ATGTAGGCTATGACGTTGCA
CYP2C19	GAAGAGGAGCATTGAGGACCG	GCCCAGGATGAAAGTGGGAT
CYP2B6	CCGGGGATATGGTGTGATCTT	CCGAAGTCCCTCATAGTGGTC
CYP2A6	GAGTTCCTGTCACTGTTGCG	GTCCTGGCAGGTGTTTCATC
CYP2C8	CATTACTGACTTCCGTGCTACAT	CTCCTGCACAAATTCGTTTTCC
UGT1A1	TAAGTGGCTACCCCAAAACG	GCTTTGCATTGTCCATCTGA
UGT1A3	TCAGATGGACAATGCAAAGCGC	GGCGCATGATGTTCTCCTTGTA
UGT1A4	AACGGGAAGCCACTATCTCA	TCAGCAATTGCCATAGCTTTC
UGT1A6	AATTTCCTAAAGGCCGGTCA	TTGATCCCAAAGAGAAAACCA
UGT2B7	AACGTAATTGCATCAGCCCT	GGTCATTCTGGGGTATCCAC
UGT2B15	GTTTTCTCTGGGGTCGATGA	ATTTGGCTTCTTGCCATCAA
NTCP	AGGTCCCCATCATAGATCCC	AGGGGGACATGAACCTCAG

OATP1B1	TTCAATCATGGACCAAAATCAA	TGAGTGACAGAGCTGCCAAG
OATP1B3	GAAAACAAGACGCTGCAATG	TCCTTTCTATTTGAGTGATGGAAA
MRP2	AGCGTCCTCTGACACTCG	GGCATCTTGGCTTTGACT
MDR1	CTAATGCCGAACACATTGGA	CAGTCGCTTTATTTCTTTGCC
MRP3	GGAGGGCATCAGGCAGGGTGA	GACACAAAGGCCTTCTCGGCGT
SLC22A1	CCCCACATTCGTCAGCGGTGT	AGGTGCCCGAGGGTTCTGAGG
AHR	CAAATCCTTCCAAGCGGCATA	CGCTGAGCCTAAGAACTGAAAG
FXR	CAGGATTTCAGACTTTGGACCAT	CTTCAACCGCAGACCCTTTC
PXR	CTCACCTCCAGGTTTGCTTC	CTCCTTGATCGATCCTTTGC
RXRA	ATGGACACCAAACATTTCCTGC	GGGAGCTGATGACCGAGAAAG
RXRB	GCAGCCCAAATGACCCTGT	CCCGCAGCAATATGACCTGA
RXRG	AGAGGACGATAAGGAAGGACC	CCATGACAAGGCACTTCTGAT
SHIP	CCCCAAGGAATATGCCTGCC	TAGGGCGAAAGAAGAGGTCCC
GR	ATAGCTCTGTTCCAGACTCAACT	TCCTGAAACCTGGTATTGCCT
LXRA	ACACCTACATGCGTCGCAAG	GACGAGCTTCTCGATCATGCC
PPARα	AGAGATTTCGCAATCCATCGG	ACTGGTATTCCGTAAAGCCAAAG

Supplementary Table 2. Antibodies used in this study.

Primary Antibody

Antibody	Company	Product Code	Ig Species	Dilution
SOX2	Abcam	ab75485	Mouse IgG1	100(IF)
OCT4	Chemico	MAB4401	Mouse IgG1	500(IF)
SOX17	R&D Systems Inc	AF1924	Goat IgG	100(IF)
FOXA2	R&D Systems Inc	AF2400	Goat IgG	100(IF)
HNF1β	Santa Cruz	sc-7411	Goat	200(IF)
HNF4α	Abcam	ab41898	Mouse IgG2a	1000(IF)
AFP	Sigma	A8452	mouse IgG2a	200(IF)
ALB	Bethyl	A80-129A	Goat	500(IF)
CK18	Santa Cruz	sc-6259	Mouse IgG1	100(IF)
Connexin 32	Sigma	C6344	Mouse IgG1	200(IF) 1000(WB)
Connexin 43	Abcam	ab11370	Rabbit	100(IF)
CPS1	Santa cruz	sc-30060	Rabbit	100(IF)
E-Cadherin	BD Biosciences	610181	Mouse IgG2a	100(IF)
p38 MAPK	CST	9212s	Rabbit	1000(WB)
Phospho-p38 MAPK (Thr180/Tyr182)	CST	9211s	Rabbit	1000(WB)
Anti-Phosphoserine/threonine antibody	Abcam	ab17464	Rabbit	1000(WB)
β-actin antibody	Cell signaling	12262	Mouse	1000(WB)

Secondary Antibody

Secondary Antibody	Company	Product Code	Dilution
Alexa Fluor® 568 Goat Anti-Mouse IgG_1 (γ1)	Invitrogen	A21124	400
Alexa Fluor® 488 Goat Anti-Mouse IgG_{2a} (γ2a)	Invitrogen	A21131	400
Alexa Fluor® 647 Goat Anti-Mouse IgG_{2b} (γ2b)	Invitrogen	A21242	400
Alexa Fluor® 647 Donkey Anti-Mouse IgG (H+L)	Invitrogen	A31571	400
Alexa Fluor® 568 Donkey Anti-Mouse IgG (H+L)	Invitrogen	A10037	400
Alexa Fluor® 488 Donkey Anti-Mouse IgG (H+L)	Invitrogen	A21202	400
Alexa Fluor® 568 Donkey Anti-Goat IgG (H+L)	Invitrogen	A11057	400
Alexa Fluor® 488 Donkey Anti-Rabbit IgG (H+L)	Invitrogen	A21206	400

Supplementary References

1. Kanehisa, M. & Goto, S. KEGG: kyoto encyclopedia of genes and genomes. *Nucleic acids research* **28**, 27-30 (2000).
2. Kanehisa, M., Sato, Y., Kawashima, M., Furumichi, M. & Tanabe, M. KEGG as a reference resource for gene and protein annotation. *Nucleic acids research* **44**, D457-462, doi:10.1093/nar/gkv1070 (2016).
